# Extraordinary anisotropic thermal expansion in photosalient crystals

**DOI:** 10.1107/S2052252519014581

**Published:** 2020-01-01

**Authors:** Khushboo Yadava, Gianpiero Gallo, Sebastian Bette, Caroline Evania Mulijanto, Durga Prasad Karothu, In-Hyeok Park, Raghavender Medishetty, Panče Naumov, Robert E. Dinnebier, Jagadese J. Vittal

**Affiliations:** a Department of Chemistry, National University of Singapore, S8-05-03, 3 Science Drive 3, 117543, Singapore; b Max Planck Institute for Solid State Research, Heisenbergstrasse 1, D70569 Stuttgart Germany; c Department of Chemistry and Biology ‘A. Zambelli’, University of Salerno, Via Giovanni Paolo II, 132, Fisciano (SA) 84084, Italy; d New York University Abu Dhabi, 129188, Abu Dhabi, United Arab Emirates

**Keywords:** solid-state reactions, [2+2] cyclo­additions, photosalient effects, thermal expansion, metal complexes, crystal engineering, mechanochemistry, properties of solids, organic solid-state reactions, molecular crystals

## Abstract

Although a plethora of metal complexes have been characterized, those having multifunctional properties are very rare. This article reports three isotypical complexes, namely [Cu(benzoate)*L*_2_], where *L* = 4-styryl­pyridine (4spy) (**1**), 2′-fluoro-4-styryl­pyridine (2F-4spy) (**2**) and 3′-fluoro-4-styryl­pyridine (3F-4spy) (**3**), which show photosalient behavior (photoinduced crystal mobility) while they undergo [2+2] cyclo­addition. These crystals also exhibit anisotropic thermal expansion when heated from room temperature to 200°C. The overall thermal expansion of the crystals is impressive, with the largest volumetric thermal expansion coefficients for **1**, **2** and **3** of 241.8, 233.1 and 285.7 × 10^−6^ K^−1^, respectively, values that are comparable to only a handful of other reported materials known to undergo colossal thermal expansion. As a result of the expansion, their single crystals occasionally move by rolling. Altogether, these materials exhibit unusual and hitherto untapped solid-state properties.

## Abstract 2

Single crystals of three isotypical Cu(II) coordination complexes with paddlewheel structures exhibit mechanical motion while undergoing [2+2] cyclo­addition under UV light (photosalient effect), and also exhibit large positive thermal expansion on heating.

## 1. Introduction   

Multifunctional smart materials can perform multiple functions through tailored chiral, electronic, magnetic, optical, thermal and/or mechanical properties that can be used for energy storage and conversion, drug delivery, catalysis, *etc*. It is relatively easy to design composite materials combining two or more solids with different properties into hybrid materials for specific applications (Ferreira *et al.*, 2016 ▸; Lu & Lieber, 2007 ▸; Abouraddy *et al.*, 2007 ▸; Wang *et al.*, 2018 ▸; Zhu & Xu, 2014 ▸; Gibson, 2010 ▸). It is, however, somewhat challenging to design a single (molecular or non-molecular) material that is capable of performing multiple functions. Nevertheless, multifunctional properties have been realized, for example, in mixed-metal oxides (Robertson *et al.*, 2015 ▸), metal–organic framework (MOFs) structures (Li *et al.*, 2016 ▸; Qiu & Zhu, 2009 ▸; Maspoch *et al.*, 2007 ▸; Cui *et al.*, 2012 ▸) and nanoparticles (Cheng *et al.*, 2012 ▸; Rolison *et al.*, 2009 ▸). Multiferroic properties have been accomplished with MOFs and metal complexes (Wu *et al.*, 2010 ▸; Ramesh & Spaldin, 2007 ▸; Spaldin *et al.*, 2005 ▸; Cheong & Mostovoy, 2007 ▸). Multifunctional properties are generally less common for discrete metal complexes or clusters. Mechanically responsive materials change their shape and size and/or move in space when activated by light, heat, pressure or chemicals (Naumov *et al.*, 2015 ▸; Sato, 2016 ▸). Among them, some *dynamic molecular crystals* undergo various movements such as curling, crawling, jumping, leaping, hopping, popping, splitting, wiggling and explosion when exposed to heat or light, phenomena known as thermosalient (TS) or photosalient (PS) effects (Nath *et al.*, 2014 ▸; Commins *et al.*, 2016 ▸). These photodynamic and thermodynamic crystals set new avenues for materials that can be used to convert light or heat into mechanical work. Anisotropic changes in their lattice parameters, accompanied by a sudden release of the accumulated strain energy, are usually considered responsible and can contribute to many of the salient effects (Naumov *et al.*, 2015 ▸; Nath *et al.*, 2014 ▸).

Recently, a great number of organic, inorganic and organometallic crystals showing these properties have been discovered (Commins *et al.*, 2016 ▸, 2015 ▸; Naumov *et al.*, 2013 ▸; Vicente *et al.*, 2016 ▸; Wang *et al.*, 2017 ▸; Hatano *et al.*, 2017 ▸; Shibuya *et al.*, 2017 ▸; Takeda & Akutagawa, 2016 ▸; Seki *et al.*, 2015 ▸; Medishetty *et al.*, 2014 ▸, 2015 ▸; Mulijanto *et al.*, 2017 ▸; Yadava & Vittal, 2019 ▸). However, the number of metal complexes showing PS or TS behavior compared with organic crystals is still rather small and limited to only a few examples (Naumov *et al.*, 2013 ▸; Sato, 2016 ▸; Nath *et al.*, 2014 ▸; Commins *et al.*, 2016 ▸). As an example of one of the prominent cases: crystals of a cobalt­(III) complex [Co(NH_3_)_5_(NO_2_)](Cl)(NO_3_) were shown to bend as well as to jump violently under UV light (Naumov *et al.*, 2013 ▸; Chizhik *et al.*, 2018 ▸). On the other hand, a thermosalient palladium(II) organometallic solid was reported to show an impressive positive and negative thermal expansion, which indicates that similar anomalous expansion could be observed in other similar materials (Panda *et al.*, 2014 ▸). A smart hybrid material was prepared by incorporating this complex into thin films of sodium caseinate which exhibits dual mechanical response (to heat and light), showing potential for preparation of hybrid materials by using salient crystals (Sahoo *et al.*, 2014 ▸). In another example, a cocrystal of probenecid and 4,4′-azo­pyridine was shown to be thermally twistable, photobendable, elastically deformable and self-healable, and thus this material can be considered a multifunctional, smart, soft crystalline solid (Gupta *et al.*, 2018 ▸). Although the discovery of such multifunctional properties in a single molecular material is very important, identification of other materials with similar properties is a rather challenging task. Here, we report that the crystals of [Cu_2_(benzoate)_4_(*L*)_2_], where *L* = 4-styryl­pyridine (4spy) (**1**), 2′-fluoro-4-styryl­pyridine (2F-4spy) (**2**) and 3′-fluoro-4-styryl­pyridine (3F-4spy) (**3**) also pop violently under UV light, and thus they are photosalient. Furthermore, crystals of these materials exhibit very large anisotropic thermal expansion when heated from room temperature to about 200°C.

## 2. Results and discussion   

### 2.1. Synthesis, single-crystal structures and photosalient behavior of **1**–**3**   

**Figure 1:**
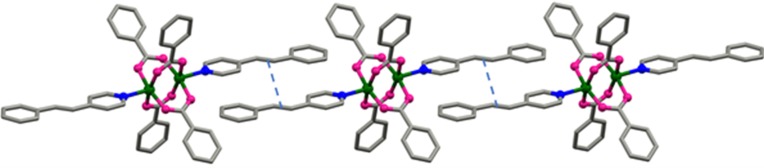
View of the one-dimensional arrangement of [Cu_2_(benzoate)_4_(4-spy)_2_] **1** via π–π interactions. Hydrogen atoms have been omitted for clarity.

Green needle-like single crystals of **1**–**3** were obtained by slow evaporation of methanol solution of Cu(NO_3_)_2_·3H_2_O, sodium benzoate and the respective pyridyl ligand (Medishetty *et al.*, 2014 ▸) in the molar ratio 1:2:1. Single-crystal X-ray diffraction (SXRD) analysis showed that they are isomorphous and isostructural to each other (see Table S1 of the supporting information) (Medishetty *et al.*, 2014 ▸). All three crystals are in the monoclinic space group *C*2/*c* with *Z* = 4, and their asymmetric unit contains half of the formula unit. A center of inversion is present in the middle of the paddlewheel structure (Fig. 1 ▸). The adjacent pyridyl ligands are stacked in a head-to-tail manner approximately normal to the (110) plane with strong *π–π* interactions between the neighboring pyridyl and phenyl groups (3.666 Å in **1**, 3.690 Å in **2** and 3.656 Å in **3**), as shown in Fig. 1 ▸. As a consequence, the centers of the C=C bonds are separated by 3.787 Å in **1**, 3.765 Å in **2** and 3.810 Å in **3**, and thus they are at distances suitable for [2+2] cyclo­addition reactions (Schmidt, 1971 ▸).

**Figure 2:**
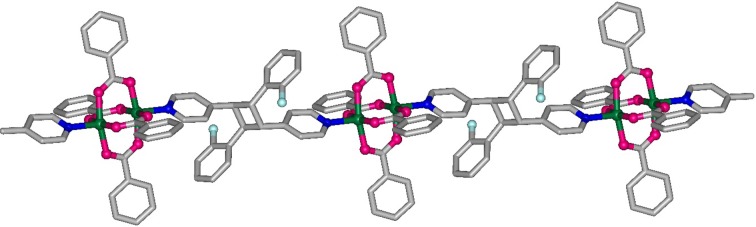
Perspective view of the photoproduct **5A** showing the formation of the one-dimensional coordination polymer. Hydrogen atoms have been omitted for clarity.

As discussed earlier, the intermolecular olefin pairs on both sides of the paddlewheel structures in **1**–**3** are juxtaposed in a head-to-tail manner and can undergo a photochemical reaction quantitatively. This arrangement is expected to yield a one-dimensional coordination polymer (CP) as the photoproduct in which the [Cu_2_(benzoate)_4_] paddlewheels are joined by the product cyclo­butane ligands (Fig. 2 ▸). The course of photoreactivity of the compound with time was followed under UV light using ^1^H NMR spectroscopy. For this purpose irradiated powder samples were taken out at different time intervals and dissolved in DMSO-*d*_6_ to record the ^1^H NMR spectra (see Figs. S15 and S18 of the supporting information). The disappearance of the olefinic protons at 8.13 p.p.m., the appearance of cyclo­butane protons at 4.82 p.p.m., and a shift in the pyridyl protons from 7.65 and 7.93 p.p.m. to 8.38 and 8.65 p.p.m., confirmed the formation of the expected cyclo­butane ring photoproduct. The other two compounds **2** and **3** also showed quantitative photoconversion of their C=C bonds to cyclo­butane rings (see Figs. S16, S17, S19 and S20). After the photoreaction of **1**–**3**, the respective one-dimensional CPs (hereafter, **4**, **5** and **6**) were semi-crystalline, as confirmed using powder X-ray diffraction (PXRD) (see Fig. S4). Crystal structure determination of a recrystallized sample of **5** provided further evidence of the formation of the one-dimensional CP [Cu_2_(benzoate)_4_(*rctt*-2F-ppcb)], where *rctt*-2F-ppcb = *rctt*-1,3-bis­(4-pyridyl)-2,4-bis­(2′-fluoro­phenyl)­cyclo­butane, (**5A**; see Fig. 2 ▸). The recrystallized photoproduct **5A** crystallizes in the space group 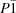 and contains the centrosymmetric binuclear paddlewheel unit connecting the cyclo­butane spacer ligand *rctt*-2F-ppcb. This result corroborated the conclusion based on the ^1^H NMR spectra (Medishetty *et al.*, 2014 ▸, 2015 ▸) that the photodimer is the only product and there are no other chemical intermediates.

Interestingly, the crystals and powders of **1**–**3** started popping violently and exploded under UV light in a similar way to popping corn on hot surfaces, which is clear evidence of the PS effect (see Movies S1–S6 of the supporting information). The single crystals, depending upon their size and shape, display different types of movements under UV light, similar to isotypical Zn(II) complexes (Medishetty *et al.*, 2014 ▸). Given the structural similarity, we conclude that the mechanism of the PS behavior of **1**–**3** is analogous to that of the respective Zn(II) complexes, which posits the existence of both reactants and photoproducts in the single crystals and rapid buildup of stress in the crystal, until they pop out or fragment into smaller pieces.

The densities of **1**–**6** were measured by the flotation method. A comparison of the densities of **5** [after complete photoreaction, 1.28 (2) g cm^−3^] and **5A** (after recrystallization, 1.489 g cm^−3^) shows that the molecules in **5A** (in the space group 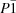) packed more tightly than in the structure of **5** after recrystallization. From other examples of photoreactive complexes, it is known that the unit cell volumes decrease upon photodimerization and formation of cyclo­butane rings. Importantly, only in the materials showing the PS effect were the unit cell volumes found to increase during the [2+2] cyclo­addition reaction (Medishetty *et al.*, 2014 ▸, 2015 ▸; Yadava & Vittal, 2019 ▸). Overall, the densities were found to decrease after photoreaction (14.7% for **1** to **4**, 15.6% for **2** to **5** and 12.2% for **3** to **6**) and these correspond to the increase in unit cell volumes of 15.4, 17.5 and 13.1% on going from **1**–**3** to **4**–**6**, respectively (Table S2). The mobility of dynamic single crystals is usually triggered by the sudden release of stress in the form of a very fast phase transition or a chemical reaction accompanied a rapid structural change that drives these phenomena (Nath *et al.*, 2014 ▸; Chizhik *et al.*, 2018 ▸; Ghosh *et al.*, 2015 ▸; Sahoo *et al.*, 2014 ▸, 2013*a* ▸,*b* ▸; Skoko *et al.*, 2010 ▸; Rawat *et al.*, 2018 ▸; Panda *et al.*, 2015 ▸, 2016 ▸; Boldyreva, 1994 ▸; Mittapalli *et al.*, 2017 ▸). Here, the stress created by the phase heterometry due to the difference in the unit cell volumes and the release of that stress manifests as motion or explosive fragmentation of the crystals.

The TS and PS effects, resulting in crystals flying over distances several times their own size, are usually associated with a very fast phase transitions, analogous to the martensitic transitions in inorganic materials (Naumov *et al.*, 2013 ▸; Nath *et al.*, 2014 ▸; Yadava & Vittal, 2019 ▸; Panda *et al.*, 2014 ▸; Ghosh *et al.*, 2015 ▸; Skoko *et al.*, 2010 ▸; Boldyreva, 1994 ▸). We observed that when heated from room temperature to 210°C, the pristine crystals of **1**–**3** occasionally rolled or jumped off the hot stage (Movies S7–S12). However, this behavior was not consistent and was not reproducible with all batches of crystals. Hence, we concluded that the motion is not a result of TS effects. Instead, it could be due to non-uniform or sudden heating. A recent report of a TS behavior of the organic compound methscopolamine bromide observed motion that was not accompanied by a detectable phase transition, and this effect was attributed to unusually large anisotropic thermal expansion with coefficients of 135 (1) × 10^−6^ K^−1^ and 114 (1) × 10^−6^ K^−1^ along the *a* and *c* axes, respectively (Klaser *et al.*, 2018 ▸). Although such behavior cannot be attributed to a TS effect (which is strictly related to a phase transition), it could nevertheless account for the observed motion of the crystals.

To obtain a better insight into the thermal behavior of the compounds reported here, we performed thermogravimetry (TG), differential scanning calorimetry (DSC) and variable temperature powder X-ray diffraction (VT-PXRD) measurements. The TG results show that **1**–**3** are thermally stable up to 210°C, and start to melt around that temperature, accompanied by decomposition to a black-colored product, probably due to formation of copper oxide along with some carbonaceous residues (Figs. S6–S8). The DSC of **1**–**3**, recorded from either single crystals or powder, did not show a phase transition from room temperature to their decomposition temperature (Figs. S12–S14). The VT-PXRD results corroborate the conclusion obtained from the DSC experiments (Figs. S29–S31).

### 2.2. Thermal expansion   

**Figure 3:**
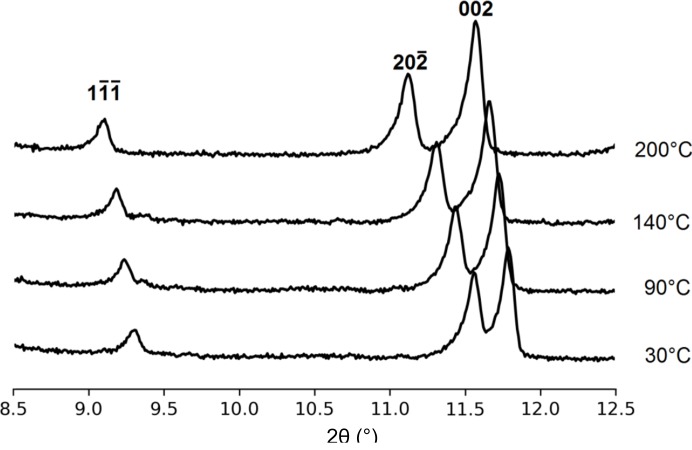
Typical PXRD pattern of **3** recorded at three different temperatures showing the shifts in selected peaks related to the thermal expansion.

The thermal behavior of crystals **1**–**3** was investigated by VT-PXRD measurements in the temperature range from room temperature to 200°C, just below their decomposition temperature (Figs. S29–S31). Since these compounds crystallize in a monoclinic space group, the coefficients of thermal expansion were calculated using the program *PASCal* (Table 1 ▸) (Cliffe & Goodwin, 2012 ▸). A typical PXRD pattern of **3** showing shifts in selected peaks related to the thermal expansion is shown in Fig. 3 ▸.

**Figure 4:**
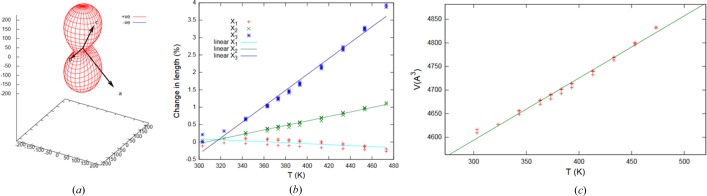
(*a*) Plot showing the variation of α with the direction, expansivity indicatrix (units: MK^−1^) in two different angles for **3**. (*b*) Overall thermal expansion of volume in **3**. (*c*) Anisotropic thermal expansion of volume in **3**.

The thermal expansion coefficients are reported along the principal *X*_2_ axis parallel to the crystallographic *b* axis, and along the principal *X*_1_ axis which is almost parallel to the direction [102] for **1** and **2** and to [101] for **3**, and along the principal *X*_3_ axis which is nearly parallel to the direction 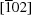 for **1** and **3** and [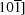 for **2**. All solids exhibit strong anisotropic thermal expansion with outstanding positive thermal expansion (PTE) along the principal *X*_3_ axis [(α_3_ = 166.38, 156.75 and 228.36) × 10^−6^ K^−1^]. Although compounds **1** and **2** show a relatively small PTE (α_1_ = 13.9159 × 10^−6^ K^−1^, α_2_ = 56.0233 × 10^−6^ K^−1^ for **1**, α_1_ = 21.8943 × 10^−6^ K^−1^, α_2_ = 38.3804 × 10^−6^ K^−1^ for **2**), compound **3** exhibits a small negative thermal expansion (α_1_ = −13.8283 × 10^−6^ K^−1^) along the principal *X*_1_ axis. The details are displayed for **3** in Fig. 4 ▸ and for **1** and **2** in Figs. S32 and S33, respectively. Furthermore, no hysteresis was observed on cooling for any of the crystals, and all expansions are rather linear in the measured temperature range [see Figs. S22–S24, (*b*) and (*c*)].

**Figure 5:**
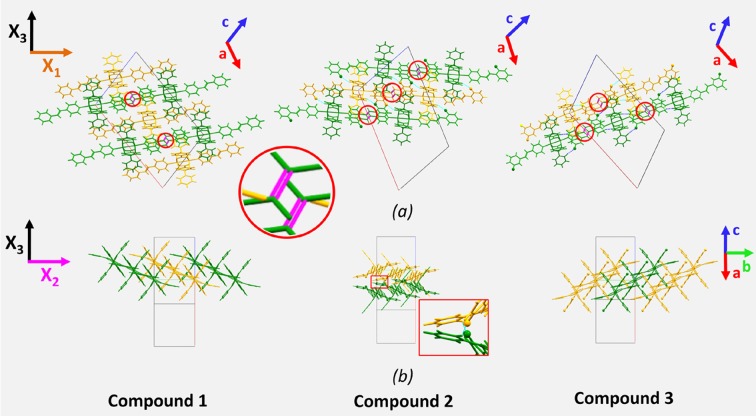
Representation of the crystal structures of compounds **1**–**3**, (*a*) along the crystallographic *b* axis and (*b*) in the [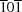] direction. The principal axes (*X*_1_, *X*_2_ and *X*_3_) for the thermal expansion are also shown. Chain-like one-dimensional structural motifs formed by π–π interactions (indicated by red circles) of the C=C double bonds of the styryl­pyridine ligands are represented in green and yellow. The inset in (*b*) highlights the interchain interactions in compound **2**.

The similarities and the small differences observed in the anisotropic thermal expansion behavior of compounds **1**–**3** can be explained through a detailed analysis of the fundamental structural motifs. In all compounds the [Cu_2_(benzoate)_2_ *L*_4_] paddlewheel complexes are connected by π*—*π interactions between the C=C bonds of the styryl­pyridine ligands, resulting in one-dimensional chain-like motifs running in the [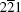] direction (Fig. 5 ▸). This direction corresponds to a combination of the principal *X*_1_ and *X*_2_ axes, in contrast to the cases reported in literature (Saha *et al.*, 2017 ▸; Saraswatula *et al.*, 2018 ▸; Crawford *et al.*, 2019 ▸), where the major expansion occurs along the π—π stacking direction; the combination of π—π interactions between the paddlewheel complexes and the strong coordination bonds in the distinct complexes strengthen the chain-like motifs inhibiting any expansion along the *X*_1_ and *X*_2_ axes.

The directive role of the π–π interactions for the thermal expansion is also confirmed by the fact that [2+2] cyclo­addition photoreactivity was observed also at higher temperatures (120–200°C). This indicates that the olefin pairs remain intact even at higher temperature, satisfying the Schmidt criteria for a [2+2] cyclo­addition reaction, *i.e.* the head-to-tail alignment of the styryl­pyridine ligands is retained when the crystals are heated. Compound **3** exhibits intra-chain F⋯H—C interactions between the fluoride-functionalized styryl­pyridine and the benzoate ligand, leading to further stiffening of the chain and could explain the slightly negative thermal expansion along the principal axis. Instead, the expansion along *X*_3_ is promoted by mechanics, which is reflected in the fact that **3** has the largest α_3_ coefficient. In **2**, F⋯H—C interactions connect neighboring chains, which additionally inhibit the thermal expansion along the principal *X*_2_ axis. Hence, **2** exhibits the smallest α_2_ coefficient of all investigated compounds. As **1** is composed only of non-substituted styryl­pyridine ligands, there are no F⋯H—C interactions, and the thermal expansion is only borne by the π–π interaction. Therefore, the determined α_1,_ α_2_ and α_3_ coefficients are in the intermediate range of the investigated compounds (Table 1 ▸). Most of the few known photosalient reactions are accompanied by chemical reactions such as [2+2] cyclo­addition or isomerization (Naumov *et al.*, 2013 ▸; Wang *et al.*, 2017 ▸; Takeda & Akutagawa, 2016 ▸; Medishetty *et al.*, 2014 ▸, 2015 ▸; Mulijanto *et al.*, 2017 ▸; Yadava & Vittal, 2019 ▸). There is also a report on the shortening of intermolecular aurophilic interactions responsible for the PS effect (Seki *et al.*, 2015 ▸). Usually the PS effect that is based on the [2+2] cyclo­addition reaction requires not only alignment of the olefin bond pairs in the solid state, but also a sudden anisotropic cell expansion during the photoreaction (Medishetty *et al.*, 2014 ▸, 2015 ▸; Mulijanto *et al.*, 2017 ▸; Yadava & Vittal, 2019 ▸). The paddlewheel metal complexes are very convenient materials to study the effects of these factors.

Complementary π–π interactions in head-to-tail alignment of the 4spy ligands are congenial to make photoreactive crystals and this alignment results in one-dimensional aggregates of the Cu(II) complexes. Furthermore , all these π–π aggregates are packed parallel to each other. Hence, the formation of the cyclo­butane rings from olefin pairs promotes anisotropic volume expansion during the photoreaction. This is further supported by the increase of the unit cell volumes of 15.4, 17.5 and 13.1% on going from **1**–**3** to **4**–**6**, respectively, as determined from the densities by the flotation method. This is corroborated by the unit cell volume measurements of **4**–**6** from XRPD experiments (Figs. S34 and S35, Tables S4 and S5). The stress generated by the phase heterometry is released suddenly in the form of a very fast chemical reaction accompanied a rapid structural change that appears to drive this PS effect.

## 3. Conclusions   

Molecular solids in general are expected to have moderate positive thermal expansion (PTE) due to increasing anharmonic vibrational amplitudes of their molecules. In many cases, structural peculiarities may give rise to very large PTE (Goodwin *et al.*, 2008 ▸; Das *et al.*, 2010 ▸, 2015 ▸; Engel *et al.*, 2014 ▸; Alimi *et al.*, 2018 ▸; Janiak *et al.*, 2018 ▸; Zhou *et al.*, 2015 ▸; Yang *et al.*, 2009 ▸) or even NTE (Chapman *et al.*, 2006 ▸; Goodwin *et al.*, 2005 ▸; Margadonna *et al.*, 2004 ▸; Pan *et al.*, 2019 ▸; Phillips *et al.*, 2008 ▸; Wu *et al.*, 2008 ▸) upon heating (Table S3). It is interesting to note that the parallel alignment of the one-dimensional assemblies in **1**–**3** promotes large thermal expansion from room temperature to 200°C in addition to photoreactivity and the PS effect. The volumetric thermal expansions (VTE) observed for **1**, **2** and **3** are 241.8, 233.1 and 285.7 × 10^−6^ K^−1^, respectively. Of these, the value observed for **3** is the largest for metal complexes, based on comparison with the previously reported value of 255.5 × 10^−6^ K^−1^ for the palladium(II) complex (Panda *et al.*, 2014 ▸). However, this is not the largest VTE reported thus far, and the difference in using different expressions to calculate thermal expansion used in the literature and the occasional non-linearity of the expansion with temperature should be considered (Table S3) (Engel *et al.*, 2014 ▸; Zhou *et al.*, 2015 ▸; Yang *et al.*, 2009 ▸). The α_3_ coefficients of the investigated compounds exceed the other thermal expansion coefficients at least by a factor of three, which leads to a progressive anisotropic expansion on heating. This creates interfacial strain in the crystals which accumulates until it is suddenly released as elastic energy and propels the crystal of its debris. When crystals of **1**–**3** were heated in the temperature range 120–200°C and illuminated under UV light they started jumping violently (similar to popcorn) while undergoing [2+2] cyclo­addition. This observation provides strong evidence that the olefin pairs are intact even at higher temperatures, thus satisfying the Schimidt criteria for a [2+2] cyclo­addition. Therefore, anisotropic expansion occurs roughly normal to the one-dimensional aggregates. We conclude that the robustness of the π–π interactions in this crystal packing is ultimately the key structural feature for all three properties observed with these materials. This work therefore provides new insights towards the engineering of multifunctional properties in crystals, and favors these and similar compounds as candidates for in-depth studies into the factors that determine the salient effects.

CCDC codes 1845040–1845043 for **1**–**3** and **5A** contain the supplementary crystallographic data for this paper. These data can be obtained free of charge from the Cambridge Crystallographic Data Centre via https://www.ccdc.cam.ac.uk/data_request/cif.

## 4. Related literature   

The following references are cited in the supporting information: Enfange *et al.* (1990 ▸); Sheldrick (1996 ▸, 2008 ▸); Müller *et al.* (2006 ▸); Yadava (2019 ▸); Bhattacharya & Saha (20143 ▸); Hutchins *et al.* (2016 ▸, 2019 ▸); Brock *et al.* (2019 ▸); Pawley (1981 ▸).

## Supplementary Material

Crystal structure: contains datablock(s) 1, 2, 3, 5a. DOI: 10.1107/S2052252519014581/lq5028sup1.cif

Supporting data. DOI: 10.1107/S2052252519014581/lq5028sup2.pdf

Video S1: photosalient effect of a single crystal of 1 showing rolling, hopping, breaking and disintegration of a single crystal during [2+2] cycloaddition reaction under UV light (Max 150 instrument, Xenon light source 150 W and wavelength of 360 nm). DOI: 10.1107/S2052252519014581/lq5028sup3.mp4

Video S2: photoalient behavior of crystal clusters of 1 under UV light. Different types of movements including hopping, breaking and disintegration of clusters of crystals are shown under UV light (Max 150 instrument, Xenon light source 150 W and wavelength of 360 nm). DOI: 10.1107/S2052252519014581/lq5028sup4.mp4

Video S3: occasional thermosalient effect exhibited by small single crystals of 1. The crystals were heated on a hot stage from room temperature to 210 degC showing wiggling, hopping and jumping movements. The video shown is four times faster than the normal speed to highlight the effect. DOI: 10.1107/S2052252519014581/lq5028sup5.mp4

Video S4: occasional thermosalient effect displayed by big crystals of 1. Small movements, rolling, hopping and sudden jumping of crystals leading to the disappearance of crystals from the sight are shown. The video shown is four times faster than the normal speed to dramatize the effect. DOI: 10.1107/S2052252519014581/lq5028sup6.mp4

Video S5: photosalient effect of 1 during [2+2] cycloaddition reaction under UV light showing the breaking and disintegration of single crystals (Max 150 instrument, Xenon light source 150 W and wavelength of 360 nm). DOI: 10.1107/S2052252519014581/lq5028sup7.mp4

Video S6: photosalient effect showing the breaking of a single crystal of 1 under UV light (Max 150 instrument, Xenon light source 150 W and wavelength of 360 nm). DOI: 10.1107/S2052252519014581/lq5028sup8.mp4

Video S7: photosalient effect showing the hopping and breaking of a single crystal of 1 under UV light (Max 150 instrument, Xenon light source 150 W and wavelength of 360 nm). DOI: 10.1107/S2052252519014581/lq5028sup9.mp4

Video S8: occasional thermosalient effect on big crystals of 1 showing chipping, rolling and hopping of crystals. DOI: 10.1107/S2052252519014581/lq5028sup10.mp4

Video S9: photosalient effect of a single crystal of 2 showing complete disintegration single crystals during [2+2] cycloaddition reaction under UV light (Max 150 instrument, Xenon light source 150 W and wavelength of 360 nm). DOI: 10.1107/S2052252519014581/lq5028sup11.mp4

Video S10: occasional thermosalient effect on big crystals of 2. Wiggling, rolling and hopping of crystals are demonstrated. DOI: 10.1107/S2052252519014581/lq5028sup12.mp4

Video S11: photosalient behavior showing breaking, rolling, hopping and jumping out of sight of single crystals of 3 during [2+2] cycloaddition reaction under UV light (Max 150 instrument, Xenon light source 150 W and wavelength of 360 nm). DOI: 10.1107/S2052252519014581/lq5028sup13.mp4

Video S12: occasional thermosalient effect on big crystals of 3 showing wiggling, rolling and hopping of crystals. DOI: 10.1107/S2052252519014581/lq5028sup14.mp4

CCDC references: 1845040, 1845041, 1845042, 1845043

## Figures and Tables

**Table:** *α* is the linear coefficient of thermal expansion, *σ_α_* is the error in the linear coefficient of thermal expansion, *a*, *b* and *c* are the projections of *X*_n_ on the unit cell axes.

**1**			Direction		
Axes	α, 10^−6^ K^−1^	σ_α_, 10^−6^ K^−1^	*a*	*b*	*c*
*X*_1_	13.9159	0.8233	0.5338	0.0000	0.8456
*X*_2_	56.0233	1.0415	0.0000	1.0000	0.0000
*X*_3_	166.3843	5.5537	0.5291	0.0000	0.8486
*V*	241.8234	6.4930			

**Table:** 

**2**			Direction		
Axes	α, 10^−6^ K^−1^	σ_α_, 10^−6^ K^−1^	*a*	*b*	*c*
*X*_1_	21.8943	0.4764	−0.3870	0.0000	−0.9221
*X*_2_	38.3804	0.5909	0.0000	−1.0000	0.0000
*X*_3_	167.7776	5.2979	0.8072	−0.0000	−0.5902
*V*	233.1210	6.2627			

**Table:** 

**3**			Direction		
Axes	α, 10^−6^ K^−1^	σ_α_, 10^−6^ K^−1^	*a*	*b*	*c*
*X*_1_	−13.8283	3.7014	0.6377	−0.0000	0.7703
*X*_2_	64.5518	1.3444	0.0000	−1.0000	0.0000
*X*_3_	228.3639	9.0008	−0.3302	0.0000	0.9439
*V*	285.6904	7.1723			

## References

[bb1] Abouraddy, A. F., Bayindir, M., Benoit, G., Hart, S. D., Kuriki, K., Orf, N., Shapira, O., Sorin, F., Temelkuran, B. & Fink, Y. (2007). *Nat. Mater.* **6**, 336–347. 10.1038/nmat1889 17471274

[bb2] Alimi, L. O., Lama, P., Smith, V. J. & Barbour, L. J. (2018). *CrystEngComm*, **20**, 631–635.

[bb108] Bhattacharya, S. & Saha, B. K. (2013). *Cryst. Growth Des.* **13**, 3299–3302.

[bb3] Boldyreva, E. (1994). *Mol. Cryst. Liq. Cryst. Sci. Technol. Sect. A. Mol. Cryst. Liq. Cryst.* **242**, 17–52.

[bb116] Brock, A. J., Whittaker, J. J., Powell, J. A., Pfrunder, M. C., Grosjean, A., Parsons, S., McMurtrie, J. C. & Clegg, J. K. (2019). *Angew. Chem. Int. Ed.* **57**, 11325–11328. 10.1002/anie.201806431 29998602

[bb5] Chapman, K. W., Chupas, P. J. & Kepert, C. J. (2006). *J. Am. Chem. Soc.* **128**, 7009–7014. 10.1021/ja060916r 16719481

[bb6] Cheng, Z., Al Zaki, A., Hui, J. Z., Muzykantov, V. R. & Tsourkas, A. (2012). *Science*, **338**, 903–910. 10.1126/science.1226338 PMC3660151 23161990

[bb7] Cheong, S.-W. & Mostovoy, M. (2007). *Nat. Mater.* **6**, 13–20. 10.1038/nmat1804 17199121

[bb8] Chizhik, S., Sidelnikov, A., Zakharov, B., Naumov, P. & Boldyreva, E. (2018). *Chem. Sci.* **9**, 2319–2335. 10.1039/c7sc04863g PMC5903420 29719705

[bb9] Cliffe, M. J. & Goodwin, A. L. (2012). *J. Appl. Cryst.* **45**, 1321–1329.

[bb10] Commins, P., Desta, I. T., Karothu, D. P., Panda, M. K. & Naumov, P. (2016). *Chem. Commun.* **52**, 13941–13954. 10.1039/c6cc06235k 27711296

[bb11] Commins, P., Natarajan, A., Tsai, C.-K., Khan, S. I., Nath, N. K., Naumov, P. & Garcia-Garibay, M. A. (2015). *Cryst. Growth Des.* **15**, 1983–1990.

[bb12] Crawford, A. W., Groeneman, R. H., Unruh, D. K. & Hutchins, K. M. (2019). *Chem. Commun.* **55**, 3258–3261. 10.1039/c9cc00836e 30810139

[bb13] Cui, Y., Yue, Y., Qian, G. & Chen, B. (2012). *Chem. Rev.* **112**, 1126–1162. 10.1021/cr200101d 21688849

[bb14] Das, D., Jacobs, T. & Barbour, L. J. (2010). *Nat. Mater.* **9**, 36–39. 10.1038/nmat2583 19935666

[bb15] Das, R. K., Aggarwal, H. & Barbour, L. J. (2015). *Inorg. Chem.* **54**, 8171–8173. 10.1021/acs.inorgchem.5b01560 26272469

[bb100] Efange, S. N., Michelson, R. H., Remmel, R. P., Boudreau, R. J., Dutta, A. K. & Freshler, A. (1990). *J. Med. Chem.* **33**, 3133–3138. 10.1021/jm00174a007 2258899

[bb16] Engel, E. R., Smith, V. J., Bezuidenhout, C. X. & Barbour, L. J. (2014). *Chem. Commun.* **50**, 4238–4241. 10.1039/c4cc00849a 24633431

[bb17] Ferreira, A. D. B. L., Nóvoa, P. R. O. & Marques, A. T. (2016). *Compos. Struct.* **151**, 3–35.

[bb18] Ghosh, S., Mishra, M. K., Ganguly, S. & Desiraju, G. R. (2015). *J. Am. Chem. Soc.* **137**, 9912–9921. 10.1021/jacs.5b05324 26192986

[bb19] Gibson, R. F. (2010). *Compos. Struct.* **92**, 2793–2810.

[bb20] Goodwin, A. L., Calleja, M., Conterio, M. J., Dove, M. T., Evans, J. S. O., Keen, D. A., Peters, L. & Tucker, M. G. (2008). *Science*, **319**, 794–797. 10.1126/science.1151442 18258911

[bb21] Goodwin, A. L., Chapman, K. W. & Kepert, C. J. (2005). *J. Am. Chem. Soc.* **127**, 17980–17981. 10.1021/ja056460f 16366530

[bb22] Gupta, P., Karothu, D. P., Ahmed, E., Naumov, P. & Nath, N. K. (2018). *Angew. Chem. Int. Ed.* **57**, 8498–8502. 10.1002/anie.201802785 29787629

[bb23] Hatano, E., Morimoto, M., Imai, T., Hyodo, K., Fujimoto, A., Nishimura, R., Sekine, A., Yasuda, N., Yokojima, S., Nakamura, S. & Uchida, K. (2017). *Angew. Chem. Int. Ed.* **56**, 12576–12580. 10.1002/anie.201706684 28834074

[bb109] Hutchins, K. M., Groeneman, R. H., Reinheimer, E. W., Swenson, D. C. & MacGillivray, L. R. (2015). *Chem. Sci.* **6**, 4717–4722. 10.1039/c5sc00988j PMC5500859 28717483

[bb110] Hutchins, K. M., Kummer, K. A., Groeneman, R. H., Reinheimer, E. W., Sinnwell, M. A., Swenson, D. C. & MacGillivray, L. R. (2016). *CrystEngComm*, **18**, 8354–8357.

[bb24] Janiak, A., Esterhuysen, C. & Barbour, L. J. (2018). *Chem. Commun.* **54**, 3727–3730. 10.1039/c8cc00952j 29589014

[bb25] Klaser, T., Popović, J., Fernandes, J. A., Tarantino, S. C., Zema, M. & Skoko, Ž. (2018). *Crystals*, **8**, 301.

[bb26] Li, B., Wen, H. -M., Cui, Y., Zhou, W., Qian, G. & Chen, B. (2016). *Adv. Mater.* **28**, 8819–8860. 10.1002/adma.201601133 27454668

[bb27] Lu, W. & Lieber, C. M. (2007). *Nat. Mater.* **6**, 841–850. 10.1038/nmat2028 17972939

[bb28] Margadonna, S., Prassides, K. & Fitch, A. N. (2004). *J. Am. Chem. Soc.* **126**, 15390–15391. 10.1021/ja044959o 15563160

[bb29] Maspoch, D., Ruiz-Molina, D. & Veciana, J. (2007). *Chem. Soc. Rev.* **36**, 770–818. 10.1039/b501600m 17471401

[bb30] Medishetty, R., Husain, A., Bai, Z., Runčevski, T., Dinnebier, R. E., Naumov, P. & Vittal, J. J. (2014). *Angew. Chem. Int. Ed.* **53**, 5907–5911. 10.1002/anie.201402040 24664890

[bb31] Medishetty, R., Sahoo, S. C., Mulijanto, C. E., Naumov, P. & Vittal, J. J. (2015). *Chem. Mater.* **27**, 1821–1829.

[bb32] Mittapalli, S., Perumalla, D. S., Nanubolu, J. B. & Nangia, A. (2017). *IUCrJ*, **4**, 812–823. 10.1107/S2052252517014658 PMC5668866 29123683

[bb33] Mulijanto, C. E., Quah, H. S., Tan, G. K., Donnadieu, B. & Vittal, J. J. (2017). *IUCrJ*, **4**, 65–71. 10.1107/S2052252516019072 PMC5331466 28250942

[bb103] Müller, P., Herbst-Irmer, R., Spek, A., Schneider, T. & Sawaya, M. (2006). *Crystal Structure Refinement: A Crystallographer’s Guide to SHELXL.* Oxford University Press.

[bb34] Nath, N. K., Panda, M. K., Sahoo, S. C. & Naumov, P. (2014). *CrystEngComm*, **16**, 1850–1858.

[bb35] Naumov, P., Chizhik, S., Panda, M. K., Nath, N. K. & Boldyreva, E. (2015). *Chem. Rev.* **115**, 12440–12490. 10.1021/acs.chemrev.5b00398 26535606

[bb36] Naumov, P., Sahoo, S. C., Zakharov, B. A. & Boldyreva, E. V. (2013). *Angew. Chem. Int. Ed.* **52**, 9990–9995. 10.1002/anie.201303757 23873664

[bb37] Pan, Z., Chen, J., Yu, R., Patra, L., Ravindran, P., Sanson, A., Milazzo, R., Carnera, A., Hu, L., Wang, L., Yamamoto, H., Ren, Y., Huang, Q., Sakai, Y., Nishikubo, T., Ogata, T., Fan, X., Li, Y., Li, G., Hojo, H., Azuma, M. & Xing, X. (2019). *Chem. Mater.* **31**, 1296–1303. 10.1021/acs.chemmater.8b04266 PMC11071054 38711569

[bb38] Panda, M. K., Centore, R., Causà, M., Tuzi, A., Borbone, F. & Naumov, P. (2016). *Sci. Rep.* **6**, 1–11. 10.1038/srep29610 PMC4941691 27403616

[bb39] Panda, M. K., Runčevski, T., Chandra Sahoo, S., Belik, A. A., Nath, N. K., Dinnebier, R. E. & Naumov, P. (2014). *Nat. Commun.* **5**, 1–8. 10.1038/ncomms5811 25185949

[bb40] Panda, M. K., Runčevski, T., Husain, A., Dinnebier, R. E. & Naumov, P. (2015). *J. Am. Chem. Soc.* **137**, 1895–1902. 10.1021/ja5111927 25581716

[bb117] Pawley, G. (1981). *J. Appl. Cryst.* **14**, 357–361.

[bb41] Phillips, A. E., Goodwin, A. L., Halder, G. J., Southon, P. D. & Kepert, C. J. (2008). *Angew. Chem. Int. Ed.* **47**, 1396–1399. 10.1002/anie.200704421 18095370

[bb42] Qiu, S. & Zhu, G. (2009). *Coord. Chem. Rev.* **253**, 2891–2911.

[bb43] Ramesh, R. & Spaldin, N. A. (2007). *Nat. Mater.* **6**, 21–29. 10.1038/nmat1805 17199122

[bb44] Rawat, H., Samanta, R., Bhattacharya, B., Deolka, S., Dutta, A., Dey, S., Raju, K. B. & Reddy, C. M. (2018). *Cryst. Growth Des.* **18**, 2918–2923.

[bb45] Robertson, L., Penin, N., Blanco-Gutierrez, V., Sheptyakov, D., Demourgues, A. & Gaudon, M. (2015). *J. Mater. Chem. C.* **3**, 2918–2924.

[bb46] Rolison, D. R., Long, J. W., Lytle, J. C., Fischer, A. E., Rhodes, C. P., McEvoy, T. M., Bourg, M. E. & Lubers, A. M. (2009). *Chem. Soc. Rev.* **38**, 226–252. 10.1039/b801151f 19088976

[bb47] Saha, B. K., Rather, S. A. & Saha, A. (2017). *Eur. J. Inorg. Chem.* **2017**, 3390–3394.

[bb48] Sahoo, S. C., Nath, N. K., Zhang, L., Semreem, M. H., Al-Tel, T. H. & Naumov, P. (2014). *RSC Adv.* **4**, 7640–7647

[bb49] Sahoo, S. C., Panda, M. K., Nath, N. K. & Naumov, P. (2013*a*). *J. Am. Chem. Soc.* **135**, 12241–12251. 10.1021/ja404192g 23875700

[bb50] Sahoo, S. C., Sinha, S. B., Kiran, M. S. R. N., Ramamurty, U., Dericioglu, A. F., Reddy, C. M. & Naumov, P. (2013*b*). *J. Am. Chem. Soc.* **135**, 13843–13850. 10.1021/ja4056323 23895677

[bb51] Saraswatula, V. G., Sharada, D. & Saha, B. K. (2018). *Cryst. Growth Des.* **18**, 52–56.

[bb52] Sato, O. (2016). *Nat. Chem.* **8**, 644–656. 10.1038/nchem.2547 27325090

[bb53] Schmidt, G. M. J. (1971). *Pure Appl. Chem.* **27**, 647–678.

[bb54] Seki, T., Sakurada, K., Muromoto, M. & Ito, H. (2015). *Chem. Sci.* **6**, 1491–1497. 10.1039/c4sc02676d PMC5811136 29560238

[bb101] Sheldrick, G. M. (1996). *SADABS*. University of Göttingen, Germany.

[bb102] Sheldrick, G. M. (2008). *Acta Cryst.* A**64**, 112–122. 10.1107/S0108767307043930 18156677

[bb55] Shibuya, Y., Itoh, Y. & Aida, T. (2017). *Chem. Asian J.* **12**, 811–815. 10.1002/asia.201700083 28220662

[bb56] Skoko, Ž., Zamir, S., Naumov, P. & Bernstein, J. (2010). *J. Am. Chem. Soc.* **132**, 14191–14202. 10.1021/ja105508b 20860383

[bb57] Spaldin, N. A. & Fiebig, M. (2005). *Science*, **309**, 391–392. 10.1126/science.1113357 16020720

[bb58] Takeda, T. & Akutagawa, T. (2016). *Chem. Eur. J.* **22**, 7763–7770. 10.1002/chem.201600794 27120702

[bb59] Vicente, A. I., Joseph, A., Ferreira, L. P., de Deus Carvalho, M., Rodrigues, V. H. N., Duttine, M., Diogo, H. P., Minas da Piedade, M. E., Calhorda, M. J. & Martinho, P. N. (2016). *Chem. Sci.* **7**, 4251–4258. 10.1039/c5sc04577k PMC6013817 30155072

[bb60] Wang, H., Bisoyi, H. K., Wang, L., Urbas, A. M., Bunning, T. J. & Li, Q. (2018). *Angew. Chem. Int. Ed.* **57**, 1627–1631. 10.1002/anie.201712781 29285875

[bb61] Wang, H., Chen, P., Wu, Z., Zhao, J., Sun, J. & Lu, R. (2017). *Angew. Chem. Int. Ed.* **56**, 9463–9467. 10.1002/anie.201705325 28626943

[bb63] Wu, S. M., Cybart, S. A., Yu, P., Rossell, M. D., Zhang, J. X., Ramesh, R. & Dynes, R. C. (2010). *Nat. Mater.* **9**, 756–761. 10.1038/nmat2803 20657590

[bb64] Wu, Y., Kobayashi, A., Halder, G. J., Peterson, V. K., Chapman, K. W., Lock, N., Southon, P. D. & Kepert, C. J. (2008). *Angew. Chem. Int. Ed.* **47**, 8929–8932. 10.1002/anie.200803925 18850600

[bb104] Yadava, K. (2019). PhD dissertation, National University of Singapore.

[bb65] Yadava, K. & Vittal, J. J. (2019). *Cryst. Growth Des.* **19**, 2542–2547.

[bb66] Yang, C., Wang, X. & Omary, M. A. (2009). *Angew. Chem. Int. Ed.* **48**, 2500–2505. 10.1002/anie.200804739 19137517

[bb67] Zhou, H.-L., Zhang, Y.-B., Zhang, J.-P. & Chen, X.-M. (2015). *Nat. Commun.* **6**, 6917. 10.1038/ncomms7917 PMC4411299 25898347

[bb68] Zhu, Q.-L. & Xu, Q. (2014). *Chem. Soc. Rev.* **43**, 5468–5512. 10.1039/c3cs60472a 24638055

