# Supplementary material for: Extraordinary anisotropic thermal expansion in photosalient crystals
Source: IUCrJ. 2020 Jan 1;7(Pt 1):83–9. doi: 10.1107/S2052252519014581 (PMC6949593; doi:10.1107/S2052252519014581)
Supplement: Supplementary file 2 [file m-07-00083-sup2.pdf]

IUCrJ

Volume 7 (2020)

Supporting information for article:

Extraordinary anisotropic thermal expansion in photosalient crystals

Khushboo Yadava, Gianpiero Gallo, Sebastian Bette, Caroline Evania Mulijanto, Durga Prasad Karothu, In-Hyeok Park, Raghavender Medishetty, Pančе Naumov, Robert E. Dinnebier and Jagadese J. Vittal

Table of Contents

1. General Experimental Section and Synthesis details
2. Crystal data
3. X-ray powder diffraction (XRPD) (simulated and bulk) (Fig.s S1-S5)
4. Thermogravimetry (Fig. S6-S11)
5. Differential Scanning Calorimetry (Fig. S12-S14)
6. Comparison of ^{1}H NMR spectra of **1,4; 2,5; and 3,6** (Fig. S15-S17)
7. Time vs photodimerization plots (Fig. S18-S20)
8. Photographs showing the photosalient effect (Fig. S21)
9. Thermal expansion plots (Fig. S22-S25)
10. Supplementary Tables

    Table S1 Selected crystallographic data

    Table S2 Changes in densities and volume in the photoreactions of **1-3**

    Table S3. Some selected coefficients of uniaxial thermal expansion (TE) and volumetric thermal expansion (VTE)
11. Packing Fig. (Fig. S26-S28)
12. Variable-temperature PXRD experiments (Fig. S29-S33)
13. Determination of Volume and densities of **4, 5** and **6** by XRPD method
14. List of Movies (Supplementary Movies 1-12)
15. References

Experimental Section

All chemicals and solvents were of reagent or better grade, were purchased from different commercial resources and used without further purification unless mentioned. The styryl pyridine ligands were synthesized using the reported literature.[1] Powder X-ray diffraction (PXRD) data were recorded on a D5005 Siemens X-ray diffractometer with graphite monochromatized CuK_α radiation (λ = 1.54056 Å) at room temperature (298 K). NMR spectra were recorded on a 400 MHz Bruker Avance 400 FT-NMR spectrometer by calibrating the residual solvent as the reference in DMSO-D_6 solution. For all NMR spectra recorded, a drop of TFA was added to dissociate the paramagnetic copper(II) from the organic ligands in **1-6**. Thermogravimetric analysis (TGA) was performed under nitrogen atmosphere with a heating rate of 5 °C min^{-1} on a TA instruments SDT-2960. Differential Scanning Calorimetry (DSC) was performed on Mettler Toledo DSC1 under nitrogen atmosphere with a heating rate of 5 °C min^{-1}. The C, H, N analysis were carried using Elementar Vario Micro Cube instrument at the Elemental Analysis Lab, CMMAC, Department of Chemistry, National University of Singapore. The UV irradiation experiments were conducted in a LUZCHEM UV reactor with a wavelength of 360 nm. In case of percentage of photoproducts formed versus time plots, the ground single crystals were packed between Pyrex glass sides placed in the UV reactor. These glass slides were flipped back at regular time intervals to maintain the uniform exposure of UV radiation or placed vertically for uniform exposure of UV light. Photosalient experiments were conducted with single crystals and powder materials using MAX150 instruments with Xenon light source 150 W with a wavelength of 360 nm. Variable-temperature X-ray powder diffraction (VT-XRPD) measurements were carried out on a Bruker D8-Advance diffractometer in Debye-Scherrer geometry (Cu$K_{\alpha1}$ radiation from a primary Ge(111)-Johann-type monochromator, Våntec detector). The samples were loaded into 0.5 mm-diameter borosilicate glass capillaries, which were spun during the measurements. The patterns for the thermal expansion analysis were collected in a 2θ range from 3° to 40° applying a total scan time of 2 hours per measurements. The temperature was adjusted using a water-cooled capillary heater (mri) and the samples were heated following the thermal cycling program (30-200-70-200-30) °C in 20 °C steps (in ranges 30-90 °C and 120-200 °C) and in 10 °C steps (in the range 90-120 °C) with a heating ramp of 2 K min^{-1}. Before each measurement, a delay time of 10 minutes was applied to ensure thermal equilibration of the sample.

1. Synthesis details

Synthesis of [Cu_2(benzoate)$_4$(4spy)$_2$], 1: Green needle-like single crystals were obtained from slow evaporation of methanolic solution of $Cu(NO_3)_2{\cdot}3H_2O$ (12.1 mg, 0.05 mmol), sodium salt of benzoic acid (14.4 mg, 0.1 mmol) and 4spy (9 mg, 0.05 mmol) and dried at room temperature. Yield: 65%. ^{1}H NMR (400 MHz), D_6-DMSO/TFA, 298 K: δ = 8.13 (d, 4H, Py-H of 4spy), 7.62-7.68 (m, 12H, 8 benzoate protons, 4 Py-H of 4spy), 7.35-7.39 (d, 2H, CH=CH of 4spy), 7.22-7.25 (m, 8H, phenyl

protons of 4spy), 7.01-7.09 (m, 14H, 12 benzoate protons, 2H of phenyl group of 4spy), 6.85-6.90 (d, 2H, CH=CH of 4spy). Elemental analysis (%) Calculated for $C_{54}H_{42}Cu_2N_2O_8$ (973.97): C, 66.59; H, 4.35; N, 2.88. Found: C, 66.37; H, 4.38; N, 2.87.

Synthesis of [Cu_2(benzoate)$_4$(2F-4spy)$_2$], 2: Green needle-like single crystals were obtained from slow evaporation of methanolic solution of $Cu(NO_3)_2{\cdot}3H_2O$ (12.1 mg, 0.05 mmol), sodium salt of benzoic acid (14.4 mg, 0.1 mmol) and 2F-4spy (10 mg, 0.05 mmol) and dried at room temperature. Yield: 67%. ^{1}H NMR (400 MHz), *D$_6$*-DMSO/TFA, 298 K: δ = 8.28- 8.29 (4H, d, Py-H of 2F-4spy), 7.73- 7.76 (m, 12H, Py-H of 2F-4spy and 8 benzoate protons), 7.55- 7.59 (d, 2H, CH=CH of 2F-4spy), 7.40- 7.43 (t, 2H, Ph-H of 2F-4spy), 7.28- 7.31 (m, 4H, Ph-H of 2F-4spy), 6.80- 7.17 (m, 16H, CH=CH of 2F-4spy, Ph-H of 2F-4spy and 12H of benzoate). Elemental analysis (%) Calculated for $C_{54}H_{40}Cu_2F_2N_2O_8$ (1009.96): C, 64.22; H, 3.99; N, 2.77. Found: C, 63.79; H, 4.09; N, 2.84.

Synthesis of [Cu_2(benzoate)$_4$(3F-4spy)$_2$], 3: Green needle-like single crystals were obtained from slow evaporation of methanolic solution of $Cu(NO_3)_2{\cdot}3H_2O$ (12.1 mg, 0.05 mmol), sodium salt of benzoic acid (14.4 mg, 0.1 mmol) and 3F-4spy (10 mg, 0.05 mmol) and dried at room temperature. Yield: 62%. ^{1}H NMR (400 MHz), *D$_6$*-DMSO/TFA, 298 K: δ = 8.19 (d, 4H, Py-H of 3F-spy), 7.66-7.70(m, 12H, 8H of benzoate and Py-H of 3F-spy), 7.32- 7.36 (d, 2H, CH=CH of 3F-spy), 7.23- 7.25 (m, 4H, Ph-H of 3F-spy), 7.10- 7.96 (m, 14 H, 12H of benzoate and 2H of Ph-H of 3F-spy), 6.87- 6.91(d, 2H, CH=CH of 3F-spy), 6.70- 6.74(t, 2H, Ph-H of 3F-spy. Elemental analysis (%) Calculated for $C_{54}H_{40}Cu_2F_2N_2O_8$ (1009.96): C, 64.22; H, 3.99; N, 2.77. Found: C, 63.76; H, 4.04; N, 2.80.

Synthesis of [Cu_2(benzoate)$_4$(*rctt*-ppcb)], 4A: Pale green single crystals were obtained by recrystallizing the UV irradiated sample of **1** in methanol and DMF by slow evaporation. Yield: 40 %. ^{1}H NMR (400 MHz), *D$_6$*-DMSO/TFA, 298 K: δ = 8.11 (d, 4H, Py-H of rctt-ppcb), 7.71-7.73 (d, 8H, benzoate protons), 7.42-7.44 (d, 4H, Py-H of *rctt*-ppcb), 7.10-7.30 (m, 14H, 12 H of benzoate, Ph-H of *rctt*-ppcb), 6.78-6.88 (m, 8H, Ph-H of *rctt*-ppcb), 4.56 (m, 4H, cyclobutane protons of *rctt*-ppcb). $C_{54}H_{42}Cu_2N_2O_8$ (973.97): C, 66.59; H, 4.35; N, 2.88. Found: C, 66.77; H, 4.37; N, 2.66.

Synthesis of [Cu_2(benzoate)$_4$(*rctt*-2F-ppcb)], 5A: Pale green single crystals were obtained by recrystallizing the UV irradiated sample of **2** in methanol and DMF by slow evaporation. Yield: 43 %. ^{1}H NMR (400 MHz), *D$_6$*-DMSO/TFA, 298 K: δ = 8.10(d, 4H, Py-H of *rctt*-2F-ppcb), 7.66- 7.67 (d, 8H, benzoate protons, 7.45- 7.47 (d, 4H, Py-H of *rctt*-2F-ppcb), 7.04- 7.24 (m, 12H, benzoate protons), 6.48- 6.88 (m, 6H, Ph-H of *rctt*-2F-ppcb), 4.63- 4.67 (m, 4H, cyclobutane protons of *rctt*-2F-ppcb). Elemental analysis (%) Calculated for $C_{54}H_{40}Cu_2F_2N_2O_8$ (1009.96): C, 64.22; H, 3.99; N, 2.77. Found: C, 63.75; H, 3.80; N, 2.94.

Synthesis of [Cu_2(benzoate)$_4$(*rctt*-3F-ppcb)], 6A: Pale green single crystals were obtained by recrystallizing the UV irradiated sample of **3** in methanol and DMF by slow evaporation. Yield: 40%. *D$_6$*-DMSO/TFA, 298 K: δ = 8.15 (d, 4H, pyridyl protons of *rctt*-3Fppcb), 7.69-7.71 (d, 8H, benzoate protons),7.44-7.46 (d, 4H, pyridyl protons of *rctt*-3Fppcb), 6.81- 7.28(m, 12H, benzoate, 6.81- 6.87 (m, 4H, phenyl protons of *rctt*-3Fppcb), 6.46-6.63 (m, 6H, phenyl protons of *rctt*-3Fppcb), 4.55 (m, 4H, cyclobutane protons of *rctt*-3Fppcb). Elemental analysis (%) Calculated for $C_{54}H_{40}Cu_2F_2N_2O_8$ (1009.96): C, 64.22; H, 3.99; N, 2.77. Found: C, 64.08; H, 3.87; N, 2.93.

2. Crystal data

Crystal data of all these crystals were collected on a Bruker APEX diffractometer attached with a CCD detector and graphite-monochromated Mo-Kα radiation ($\lambda = 0.71073$ Å) using a sealed tube. Absorption corrections were made with the program SADABS[1] and the crystallographic package SHELXTL[2,3] was used for all calculations. CCDC numbers 1845040-1845043 belong to **1**, **2**, **3** and **5A** respectively.

1 at 100(2) K: $C_{54}H_{42}Cu_2N_2O_8$, $FW = 973.97$, monoclinic, $C2/c$; $a = 24.633(11)$ Å, $b = 12.093(4)$ Å, $c = 15.509(6)$ Å, $\alpha = 90°$, $\beta = 108.480(12)°$, $\gamma = 90°$, $V = 4381(3)$ Å^3, $Z = 4$, $\rho_{calc} = 1.477$ g·cm^{-3}, $\mu = 1.032$ mm^{-1}, $GOF = 1.030$, final $R_1 = 0.0335$, $wR_2 = 0.0821$ [for 4531 data $I > 2\sigma(I)$].

2 at 100(2) K: $C_{54}H_{40}Cu_2F_2N_2O_8$, $FW = 1009.96$, monoclinic, $C2/c$; $a = 24.9699(8)$ Å, $b = 12.1074(4)$ Å, $c = 15.4675(4)$ Å, $\alpha = 90°$, $\beta = 108.809(1)°$, $\gamma = 90°$, $V = 4426.4(2)$ Å^3, $Z = 4$, $\rho_{calc} = 1.516$ g·cm^{-3}, $\mu = 1.031$ mm^{-1}, $GOF = 1.025$, final $R_1 = 0.0309$, $wR_2 = 0.0731$ [for 4396 data $I > 2\sigma(I)$].

3 at 100(2) K: $C_{54}H_{40}Cu_2F_2N_2O_8$, $FW = 1009.96$, monoclinic, $C2/c$; $a = 25.1802(18)$ Å, $b = 12.0004(9)$ Å, $c = 15.5976(10)$ Å, $\alpha = 90°$, $\beta = 108.802(2)°$, $\gamma = 90°$, $V = 4461.7(5)$ Å^3, $Z = 4$, $\rho_{calc} = 1.504$ g·cm^{-3}, $\mu = 1.023$ mm^{-1}, $GOF = 1.126$, final $R_1 = 0.0278$, $wR_2 = 0.0738$ [for 4934 data $I > 2\sigma(I)$].

5A at 100(2) K: $C_{54}H_{40}Cu_2F_2N_2O_8$, $FW = 1009.96$, triclinic, $P\bar{1}$; $a = 10.3725(5)$ Å, $b = 11.0398(5)$ Å, $c = 12.0294(5)$ Å, $\alpha = 71.354(1)°$, $\beta = 66.229(1)°$, $\gamma = 65.356(1)°$, $V = 1126.61(9)$ Å^3, $Z = 1$, $\rho_{calc} = 1.489$ g·cm^{-3}, $\mu = 1.013$ mm^{-1}, $GOF = 1.069$, final $R_1 = 0.0283$, $wR_2 = 0.0675$ [for 4010 data $I > 2\sigma(I)$].

3. PXRD (simulated and bulk)

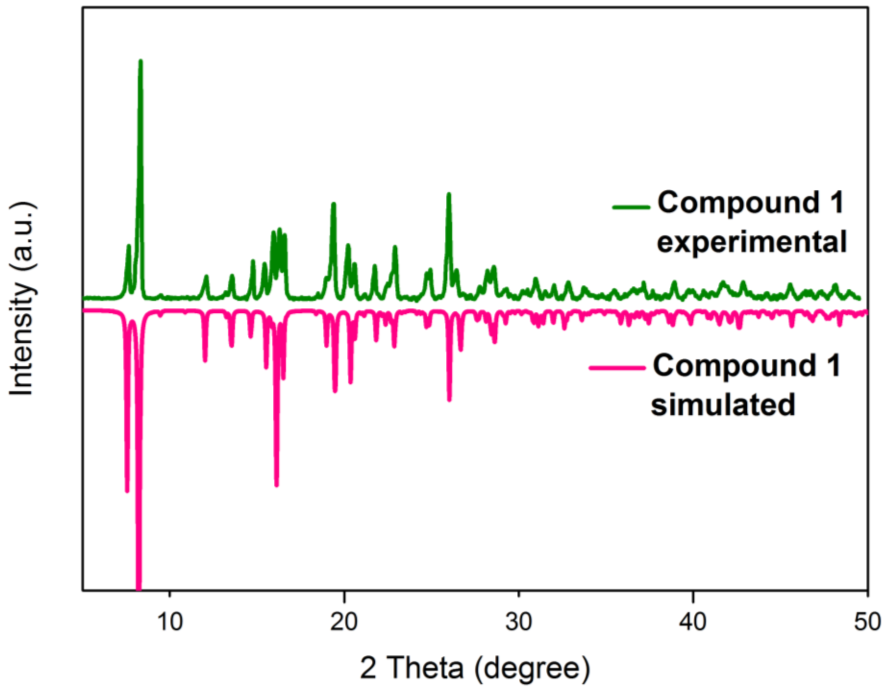

Figure S1 Comparison of the PXRD patterns of the bulk solid with the single crystal data of compound **1**.

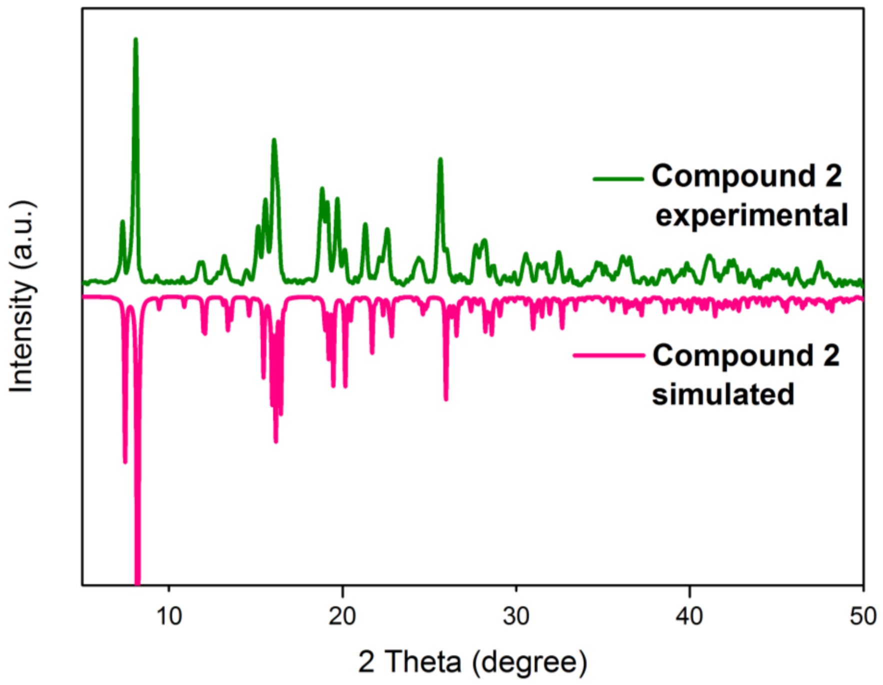

Figure S2 Comparison of PXRD patterns of the bulk solid with the single crystal data of compound **2**.

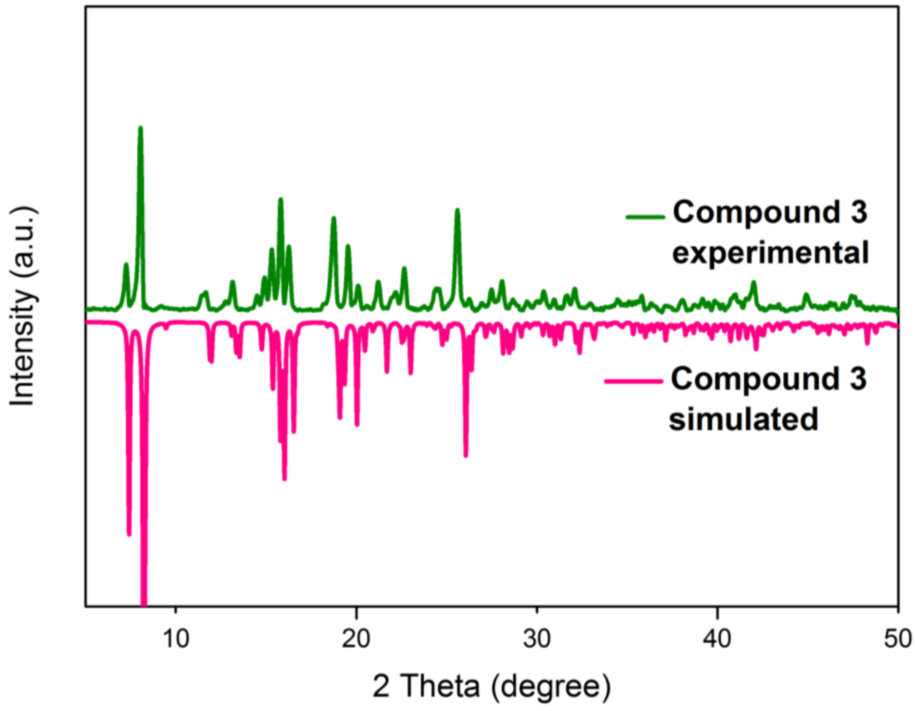

Figure S3 Comparison of PXRD patterns of the bulk solid with the single crystal data of compound **3**.

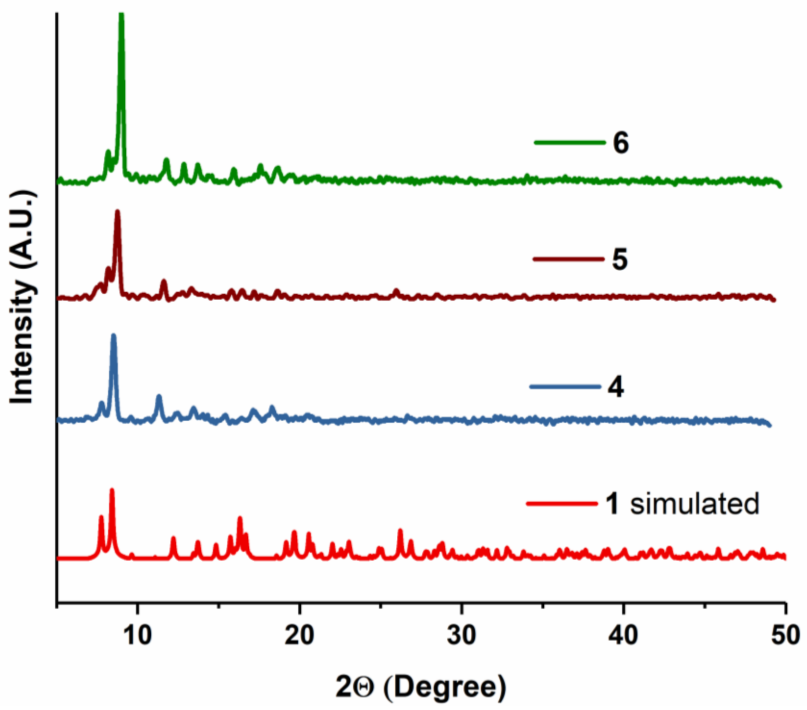

Figure S4 Powder XRD patterns of **4, 5** and **6** obtained after UV-irradiation of powdered solids of **1, 2** and **3**.

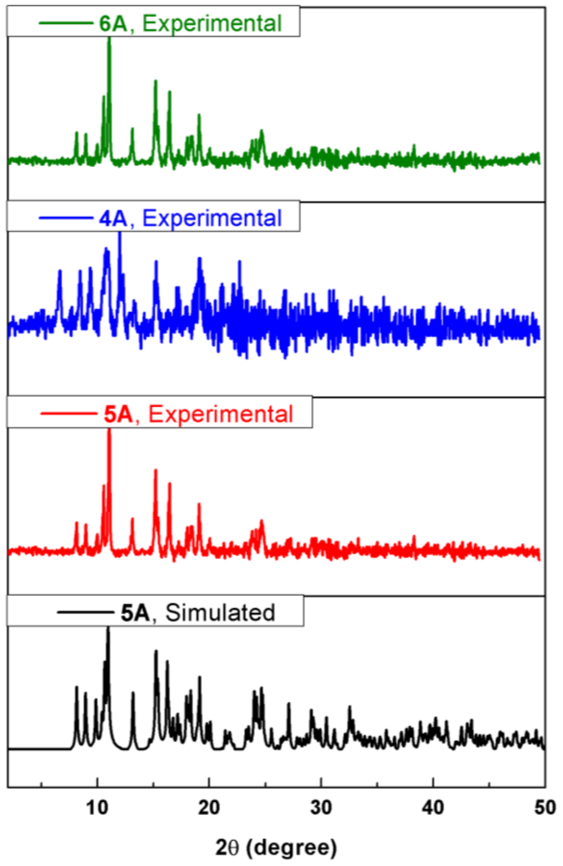

Figure S5 Powder XRD patterns of **4A, 5A** and **6A** obtained after recrystallization of completely dimerized solids of **1, 2** and **3**. It is noted that the PXRD of **4A** is different from **5A** and **6A** probably crystallized in a different space group.

4. Thermogravimetry

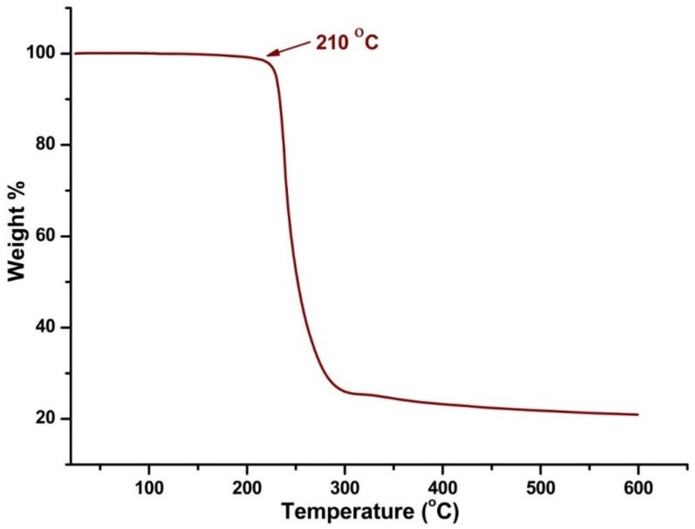

Figure S6 TGA of **1**.

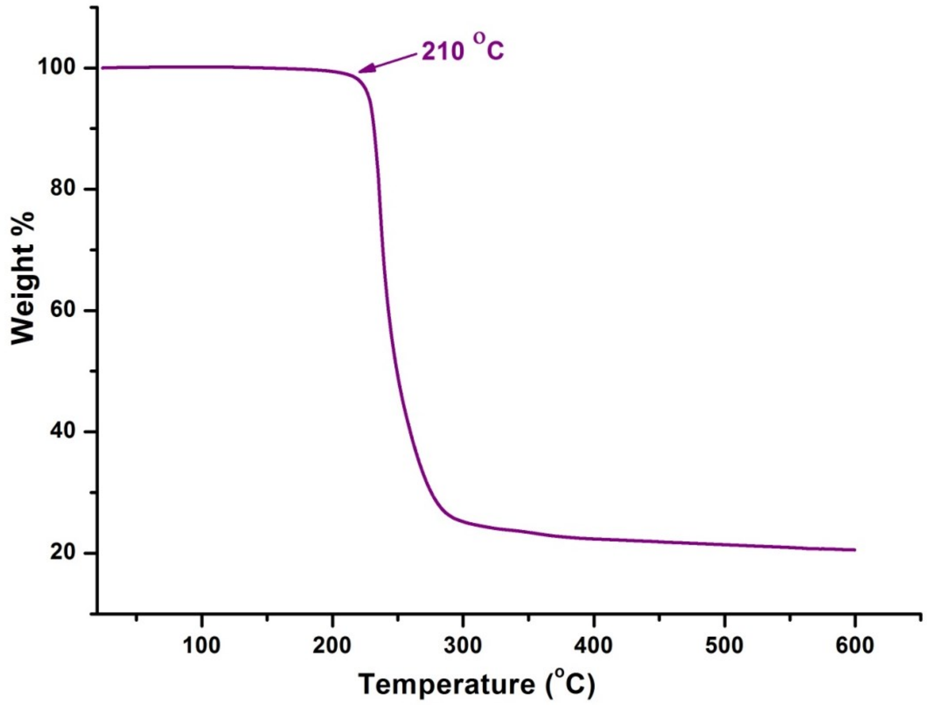

Figure S7 TGA of **2**.

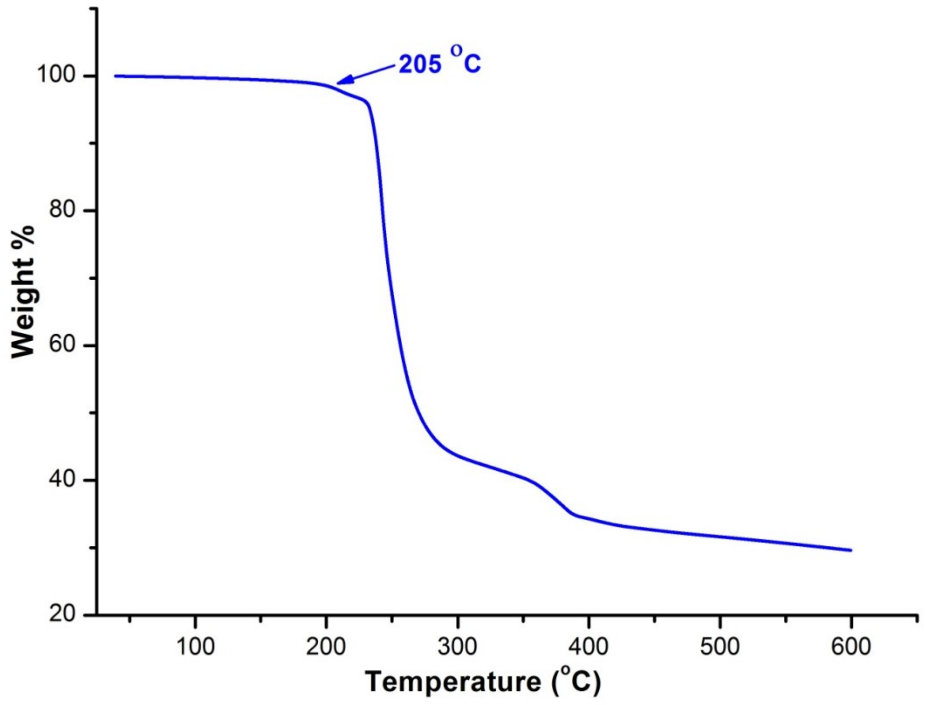

Figure S8 TGA of **3**.

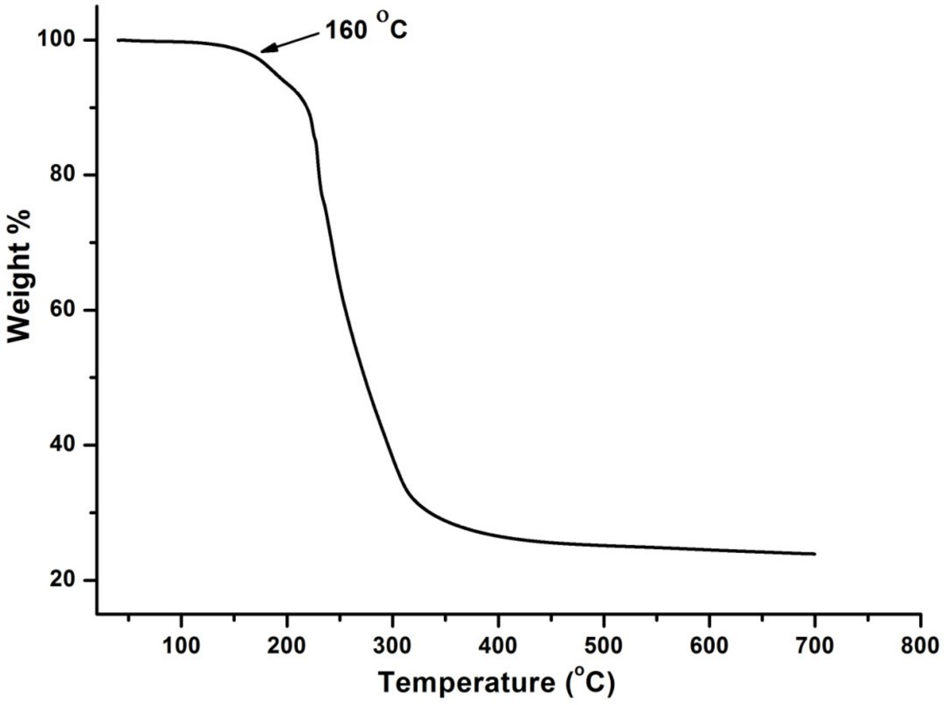

Figure S9 TGA of **4**.

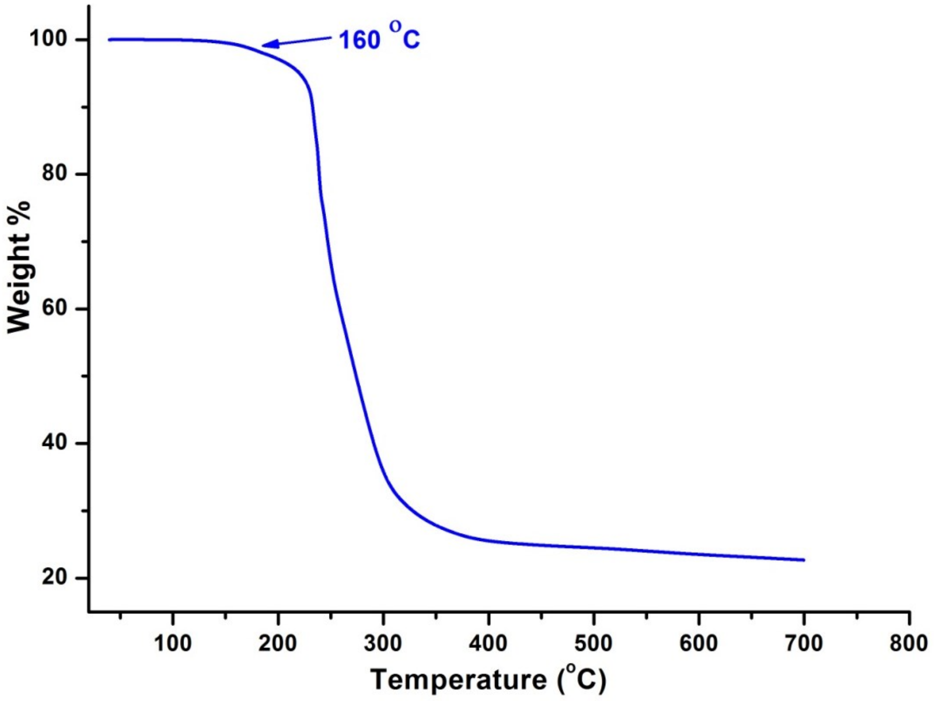

Figure S10 TGA of **5**.

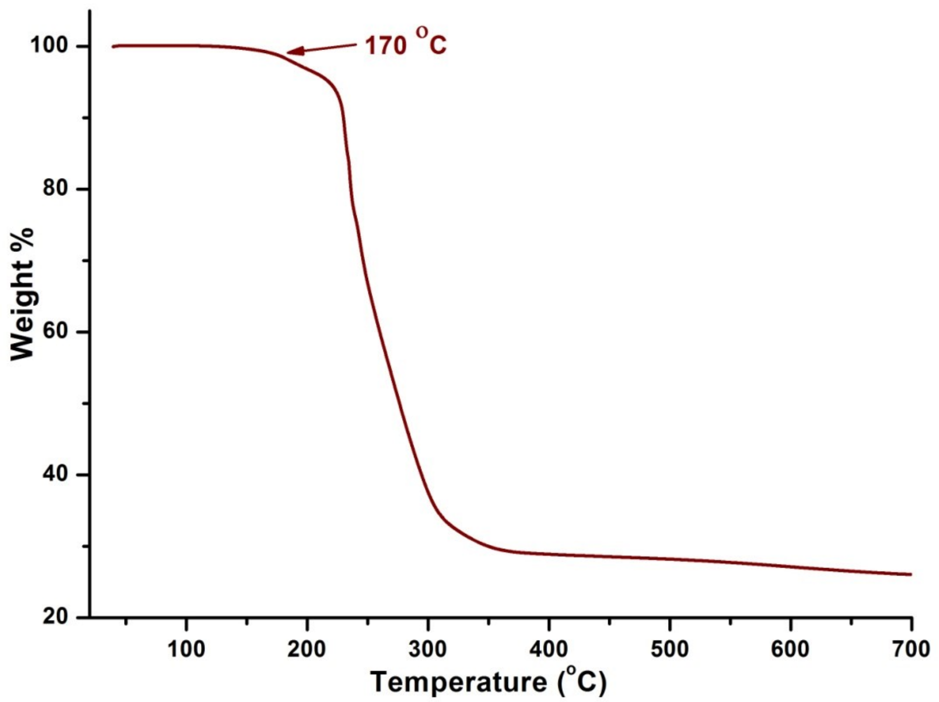

Figure S11 TGA of **6**.

5. Differential Thermal Calorimetry

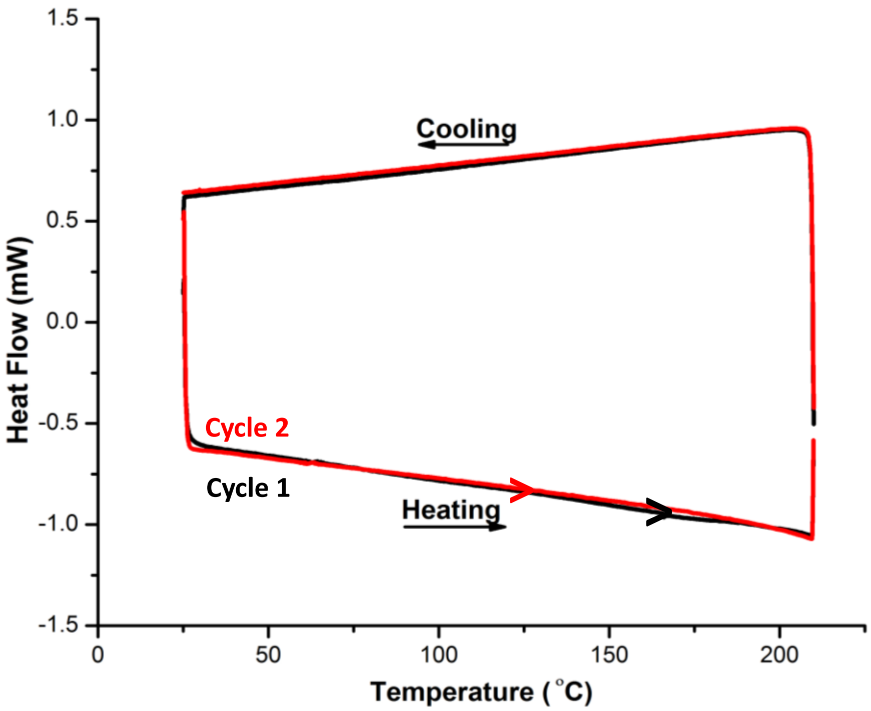

(a)

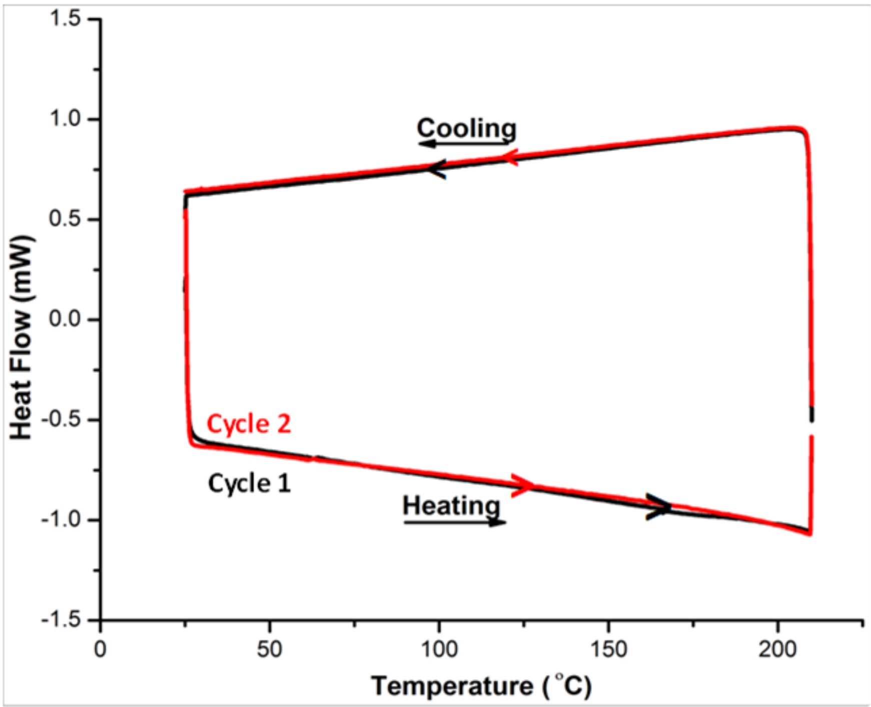

(b)

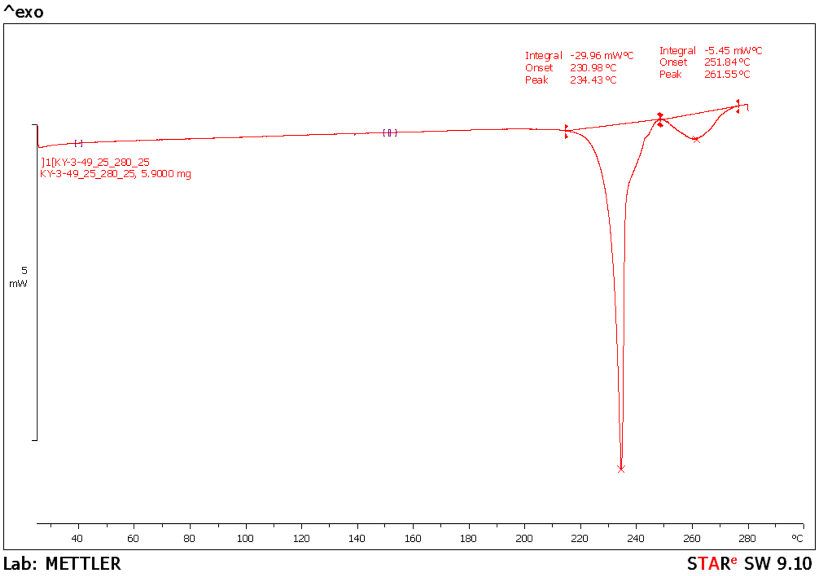

(c)

Figure S12 DSC of **1**. (a) Heating and cooling cycles before decomposition of ground powder (b) Heating and cooling cycles before decomposition of pristine crystals and (c) DSC run up to decomposition.

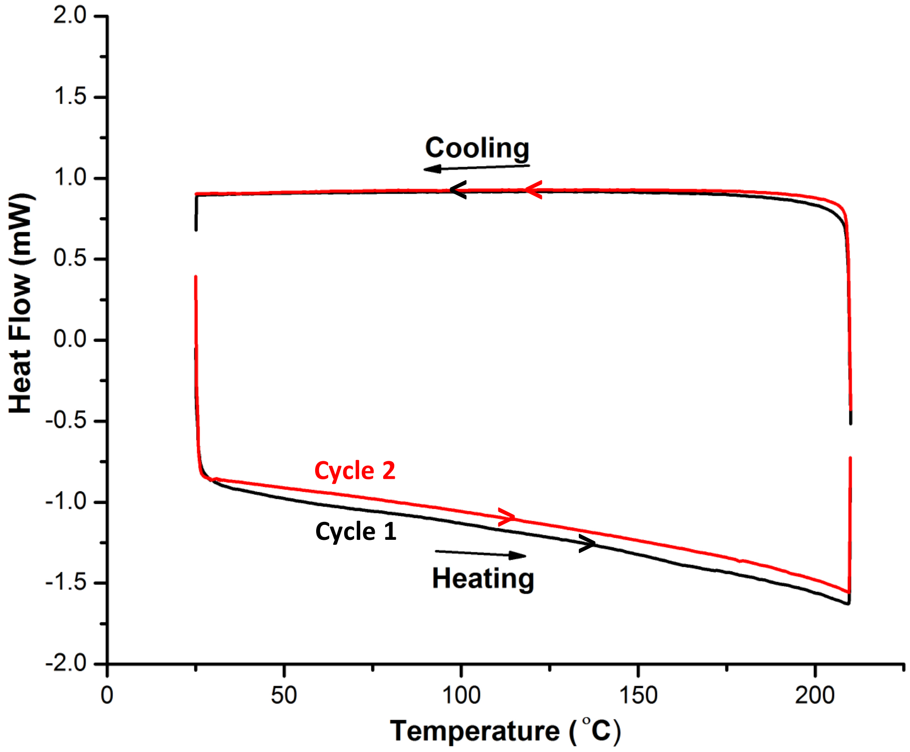
Cooling
Cycle 2
Cycle 1
Heating
Heat Flow (mW)
Temperature (°C)
2.0
1.5
1.0
0.5
0.0
-0.5
-1.0
-1.5
-2.0
0
50
100
150
200

(a)

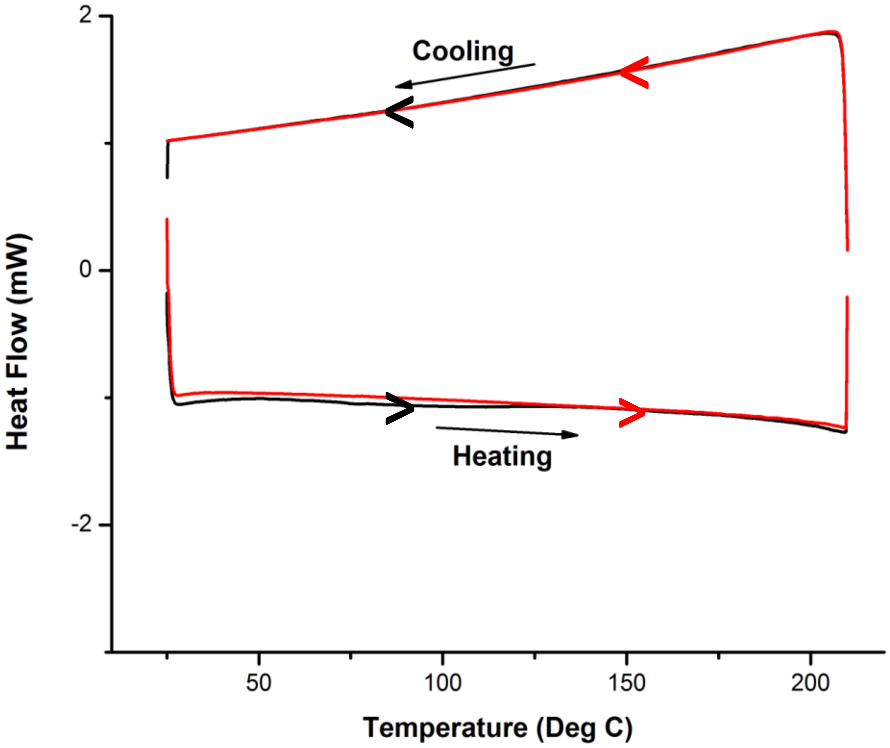
Cooling
Heating
Heat Flow (mW)
Temperature (Deg C)
2
0
-2
50
100
150
200

(b)

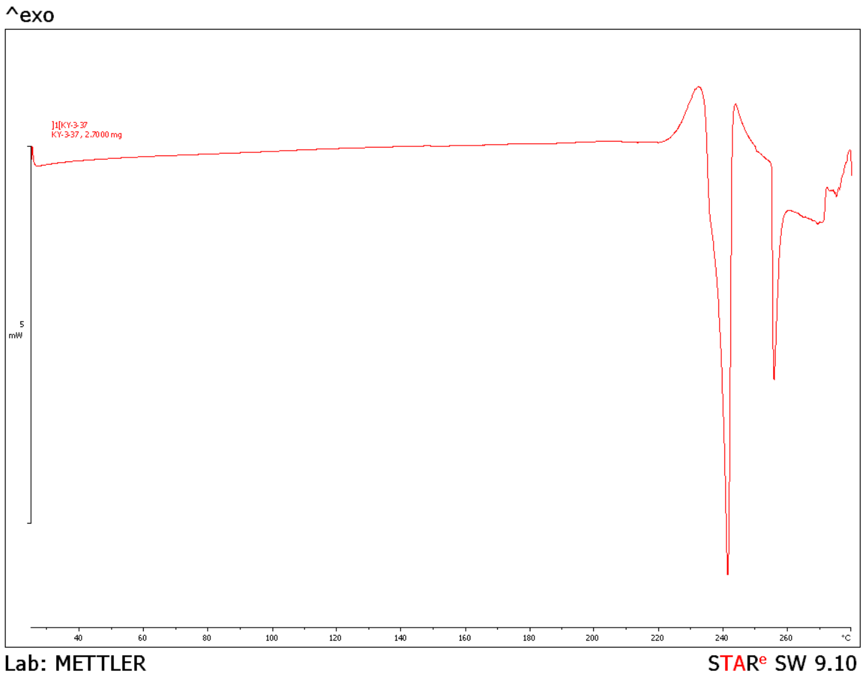

(c)

Figure S13 DSC of **2**. (a) Heating and cooling cycles before decomposition of ground powder. (b) Heating and cooling cycles before decomposition of pristine crystals and (c) DSC run up to decomposition.

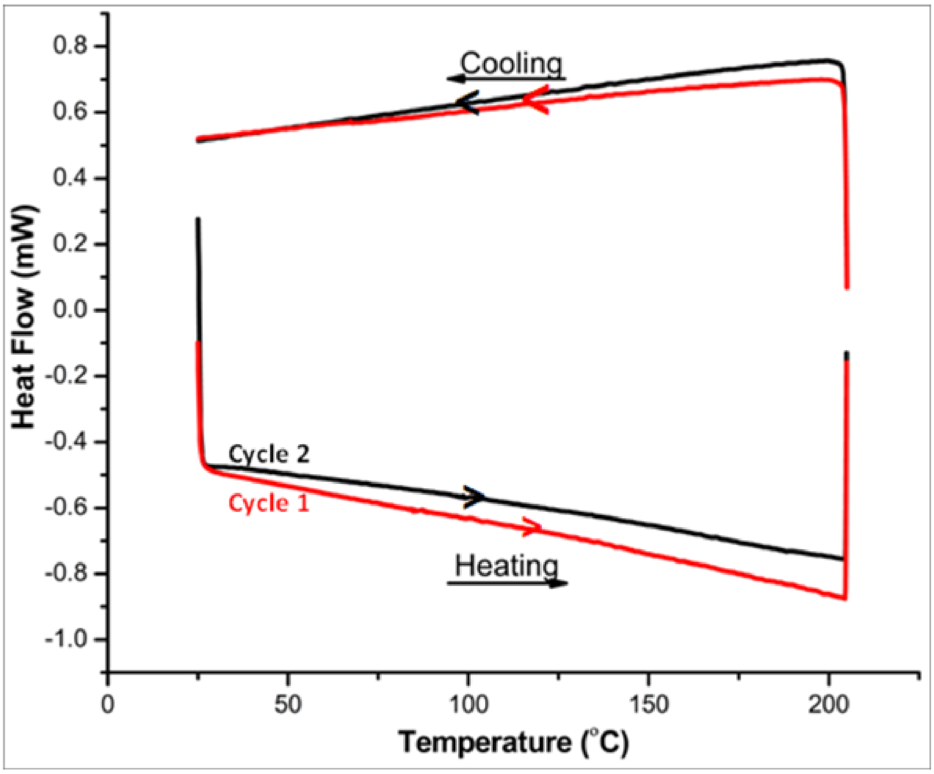
Cooling
Cycle 2
Cycle 1
Heating
Heat Flow (mW)
Temperature (°C)
0.8
0.6
0.4
0.2
0.0
-0.2
-0.4
-0.6
-0.8
-1.0
0
50
100
150
200

(a)

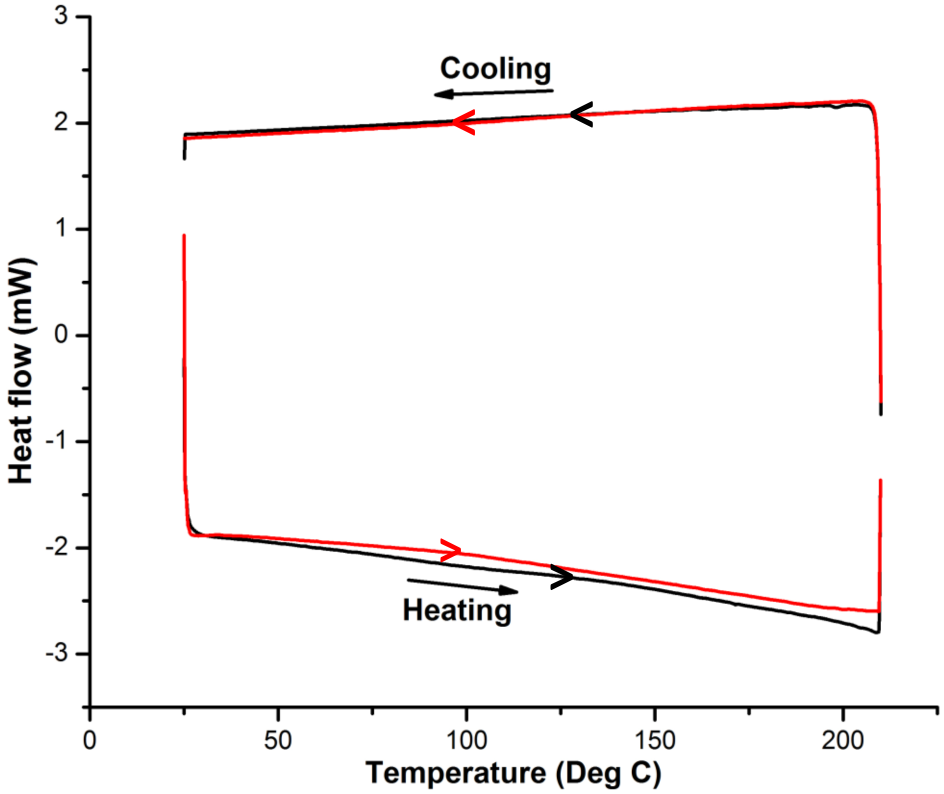
Cooling
Heating
Heat flow (mW)
Temperature (Deg C)
3
2
1
0
-1
-2
-3
0
50
100
150
200

(b)

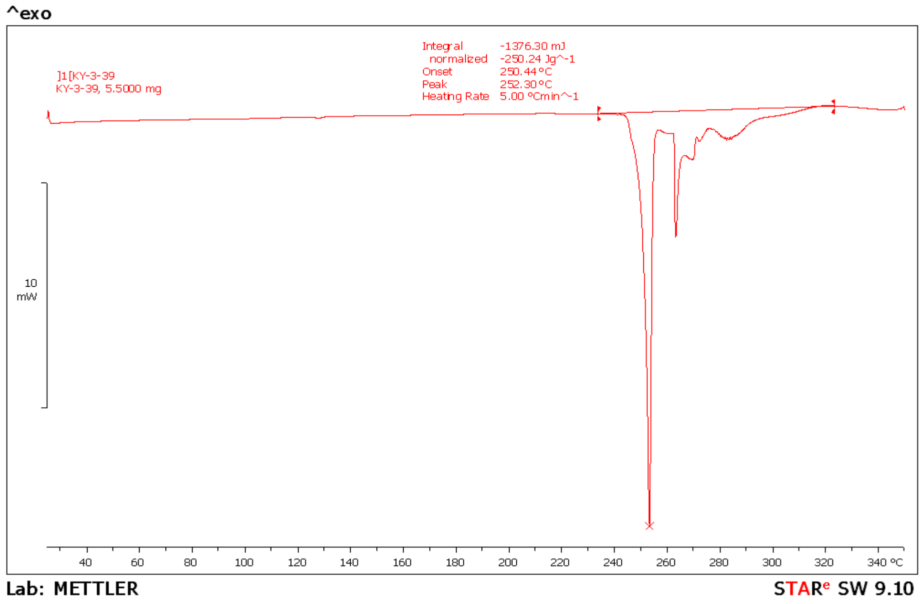

(c)

Figure S14 DSC of **3**. (a) Heating and cooling cycles before decomposition of ground powder (b) Heating and cooling cycles before decomposition of pristine crystals and (c) DSC run up to decomposition.

6. Comparison of ^{1}H NMR spectra of 1,4; 2,5; and 3,6

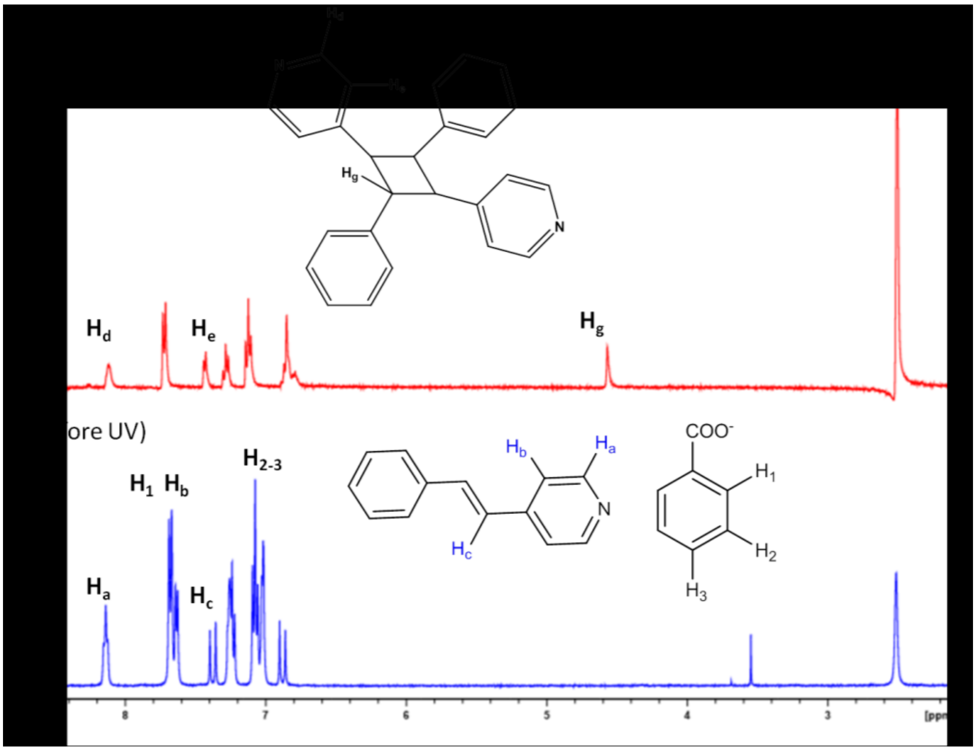

Figure S15 Stacked ^{1}H NMR spectra of **1** and **4** for a comparison of chemical shifts.

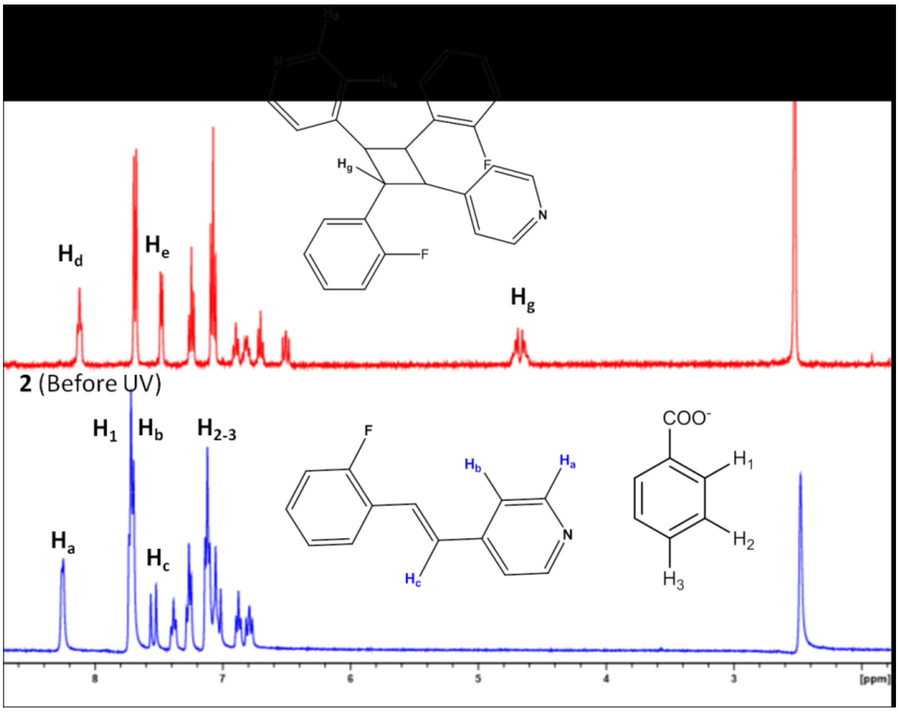

Figure S16 ^{1}H NMR spectra of **2** and **5** for a comparison of chemical shifts.

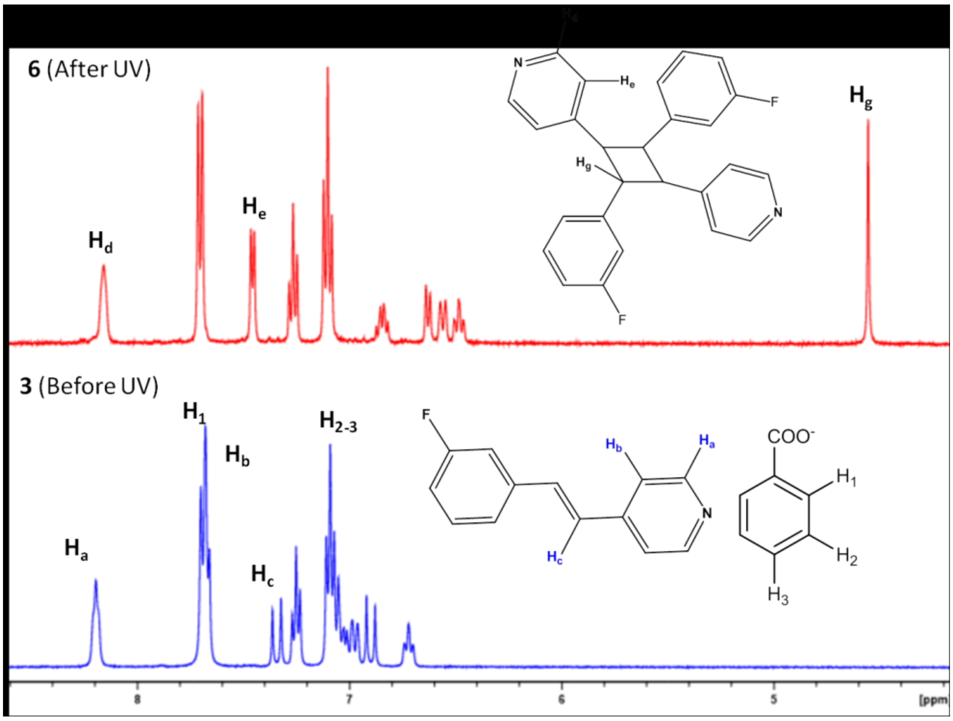

Figure S17 ^{1}H NMR spectra of **3** and **6** for a comparison of chemical shifts.

7. Time vs photodimerization plots

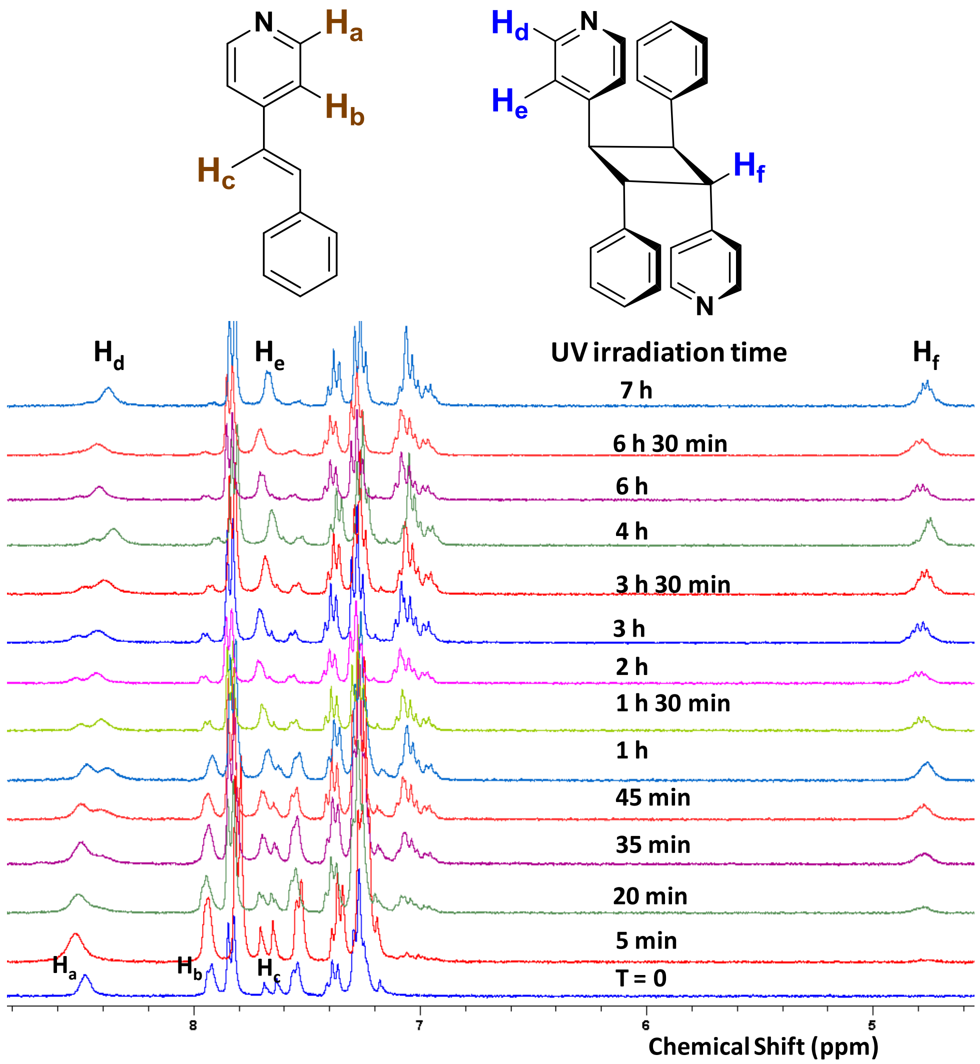

(a)

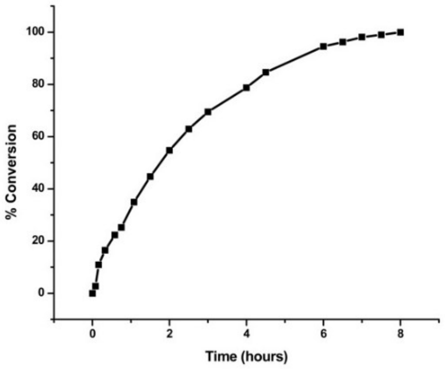

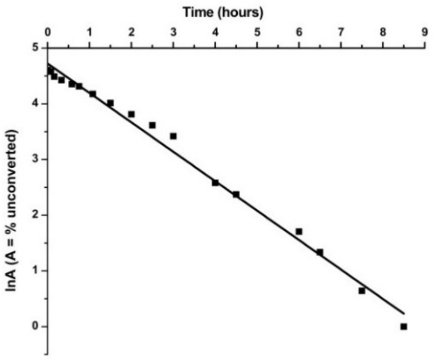

(b) (c)

Figure S18 (a) Photocycloaddition reaction of **1** monitored by ^{1}H-NMR for 8 hours. (b) Time-dimerization plot of percentage dimerized product over time. (c) Graph of ln A vs time. The rate constant was calculated to be 0.52 hour^{-1}.

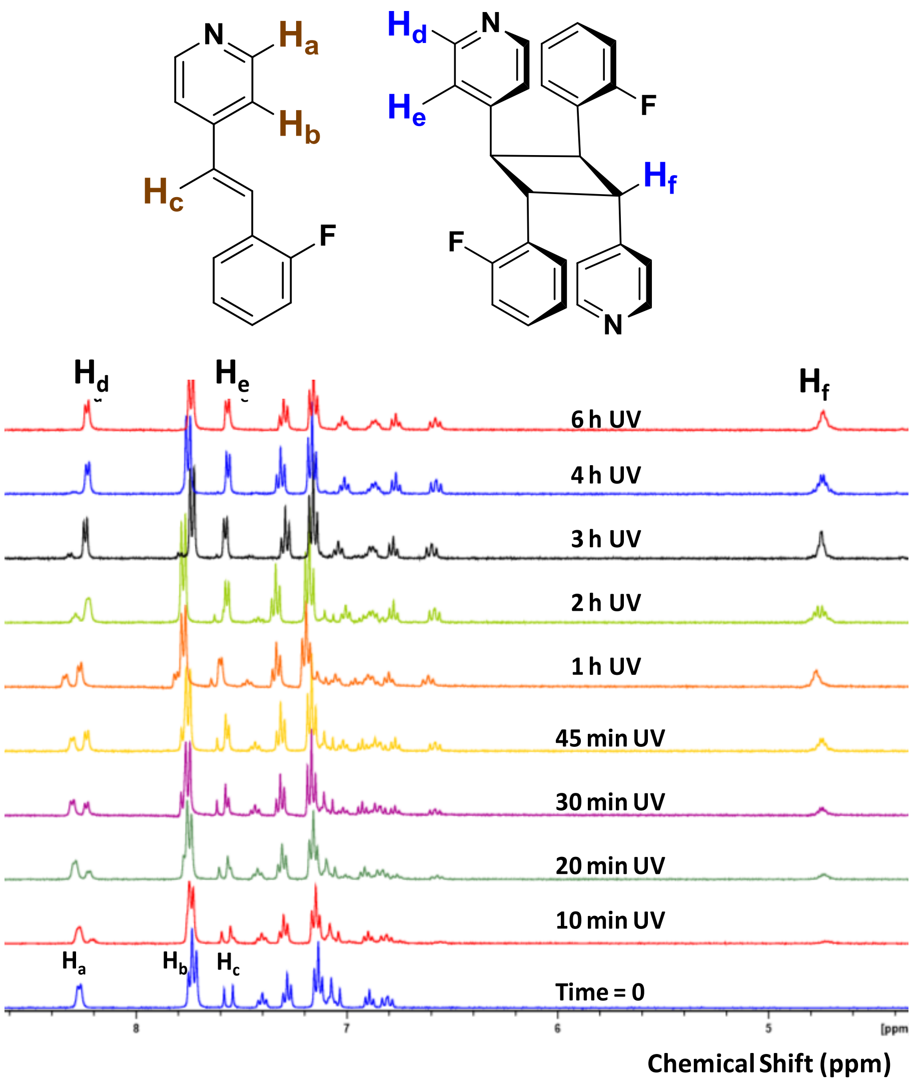

(a)

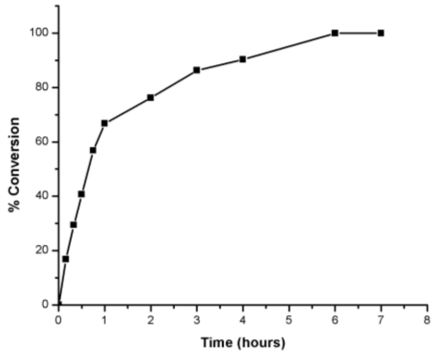

(b)

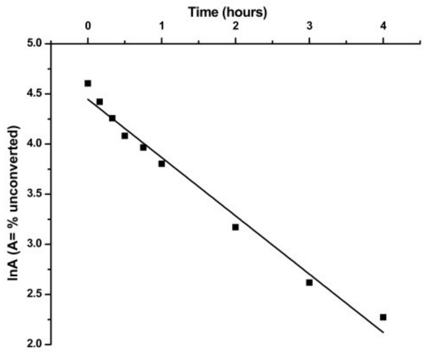

(c)

Figure S19 (a) Photo-cycloaddition reaction of **2** monitored by ^{1}H-NMR for 5 hours. (b) Time-dimerization plot of percentage dimerized product over time. (c) Graph of ln A vs time. The rate constant was calculated to be 0.58 hour^{-1}.

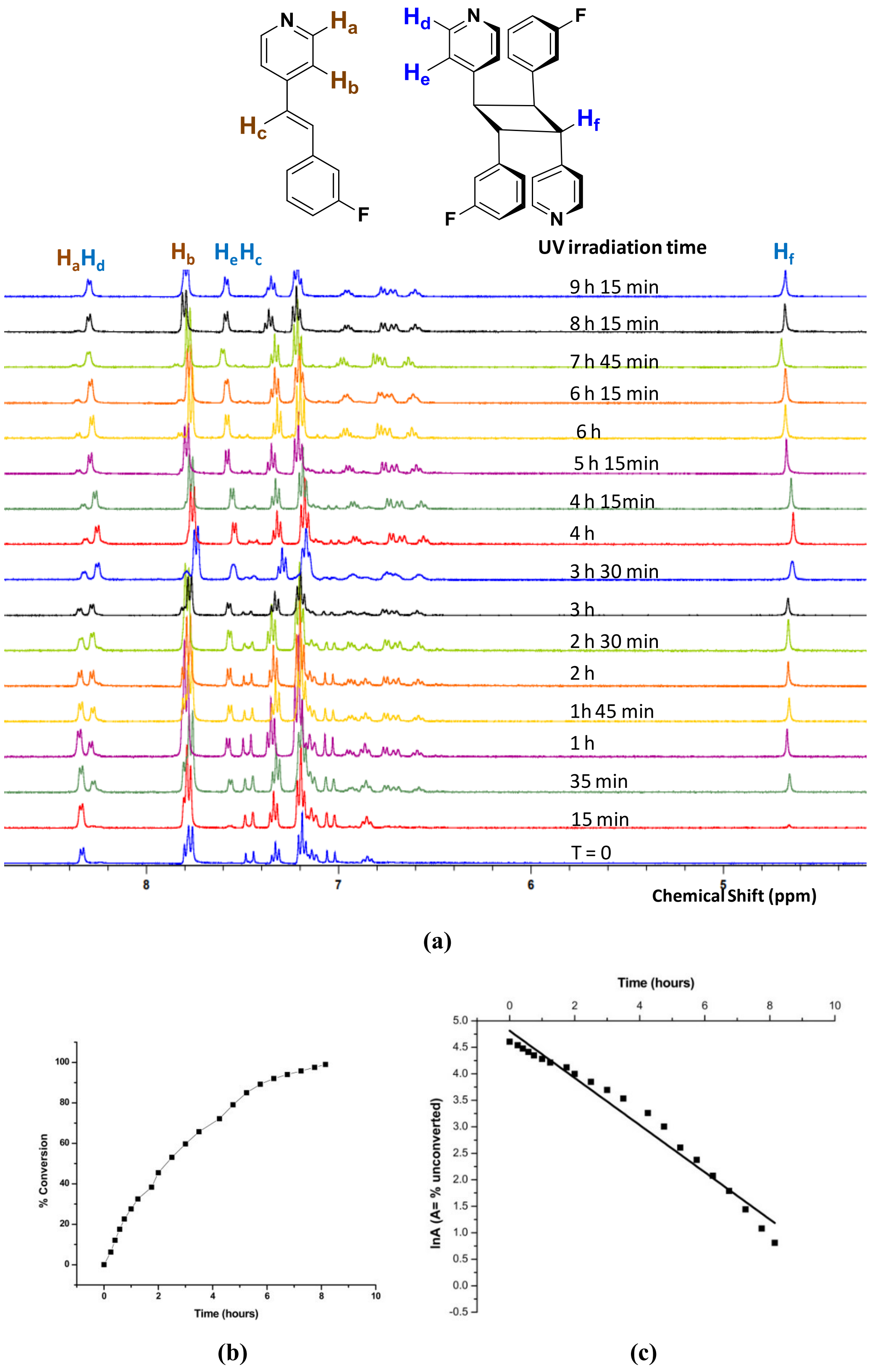

Figure S20 (a) Photo-cycloaddition reaction of **3** monitored through ^{1}H-NMR for 10 hours. (b) Time-dimerization plot of percentage dimerized product over time. (c) Graph of ln A vs time. The rate constant was calculated to be 0.44 hour^{-1}.

8. Photographs showing the photosalient effect

**Compound 1 (Before UV)**	**During/After UV**
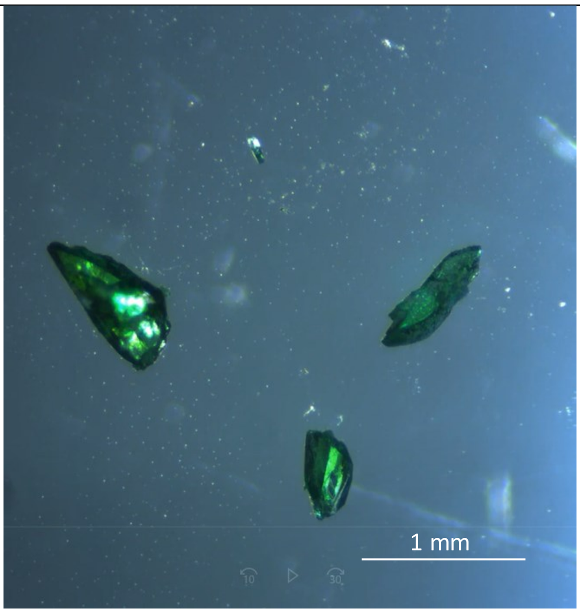	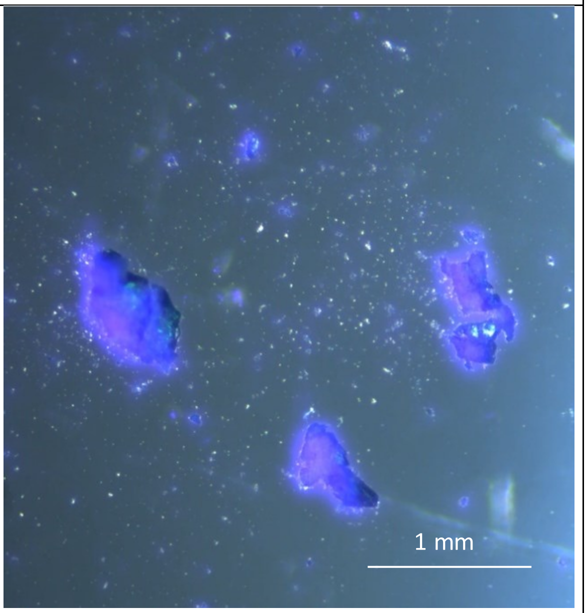
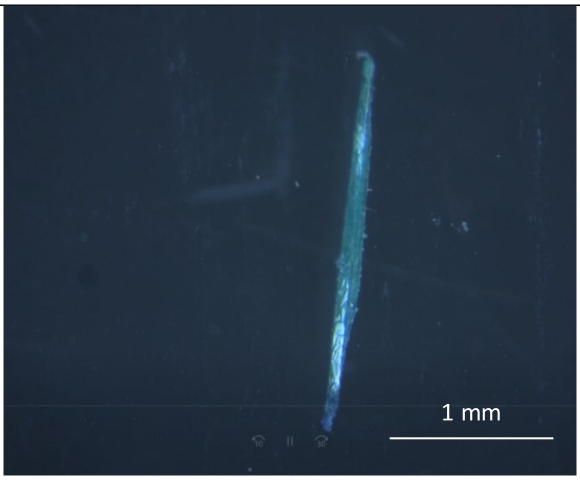	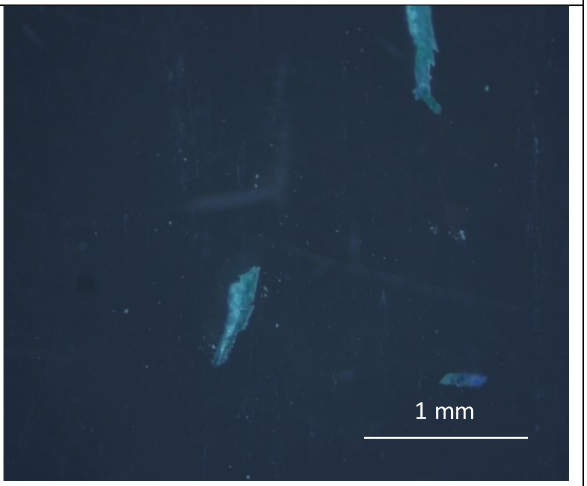

Figure S21 Before and after UV irradiation images of crystals of **1**.

**Compound 2 (Before UV)**	**After UV**
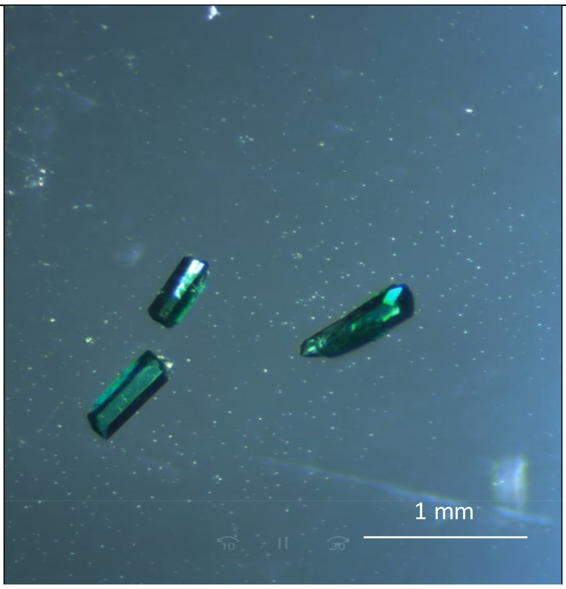	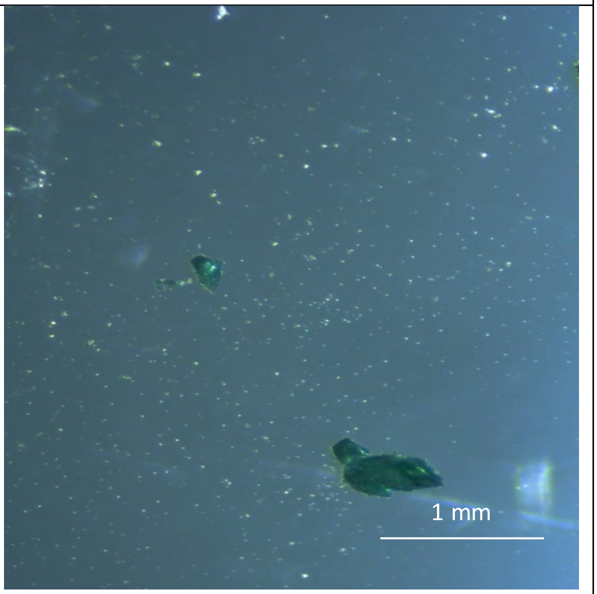

**Compound 3 (Before UV)**	**After UV**
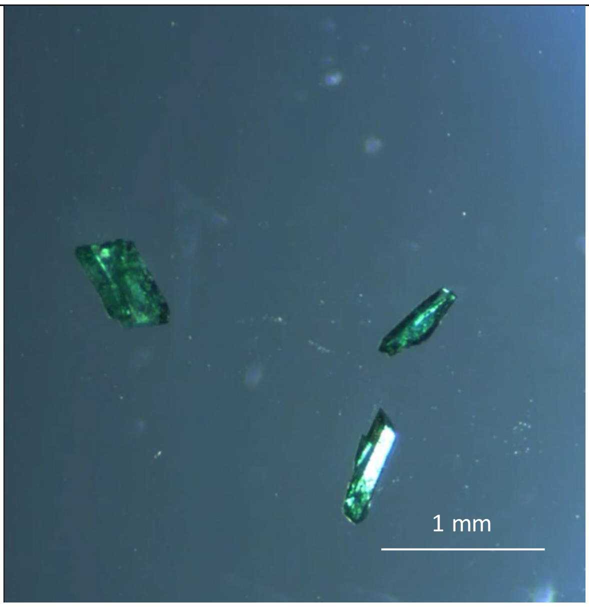	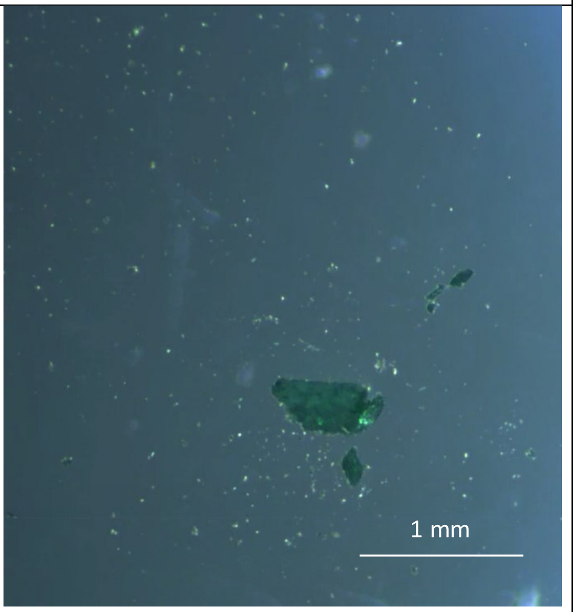

Figure S21 continued Before and after UV irradiation images of crystals of **2** and **3**.

9. Thermal Expansion Plots

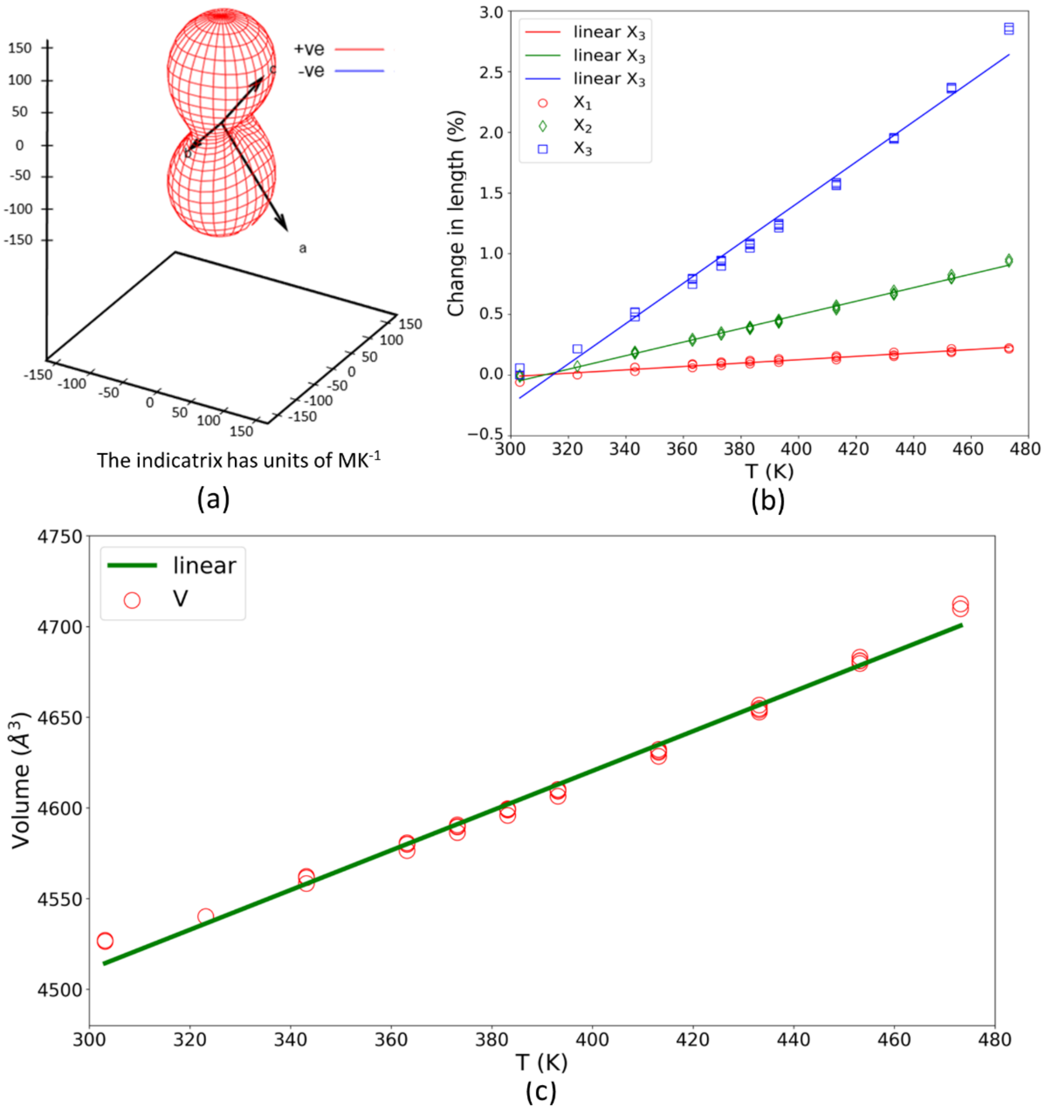

Figure S22 (a) A plot showing the variation of α with the direction, expansivity indicatrix, MK^{-1} in **1**. (b) Overall thermal expansion of volume in **1**. (c) Anisotropic thermal expansion of volume in **1**.

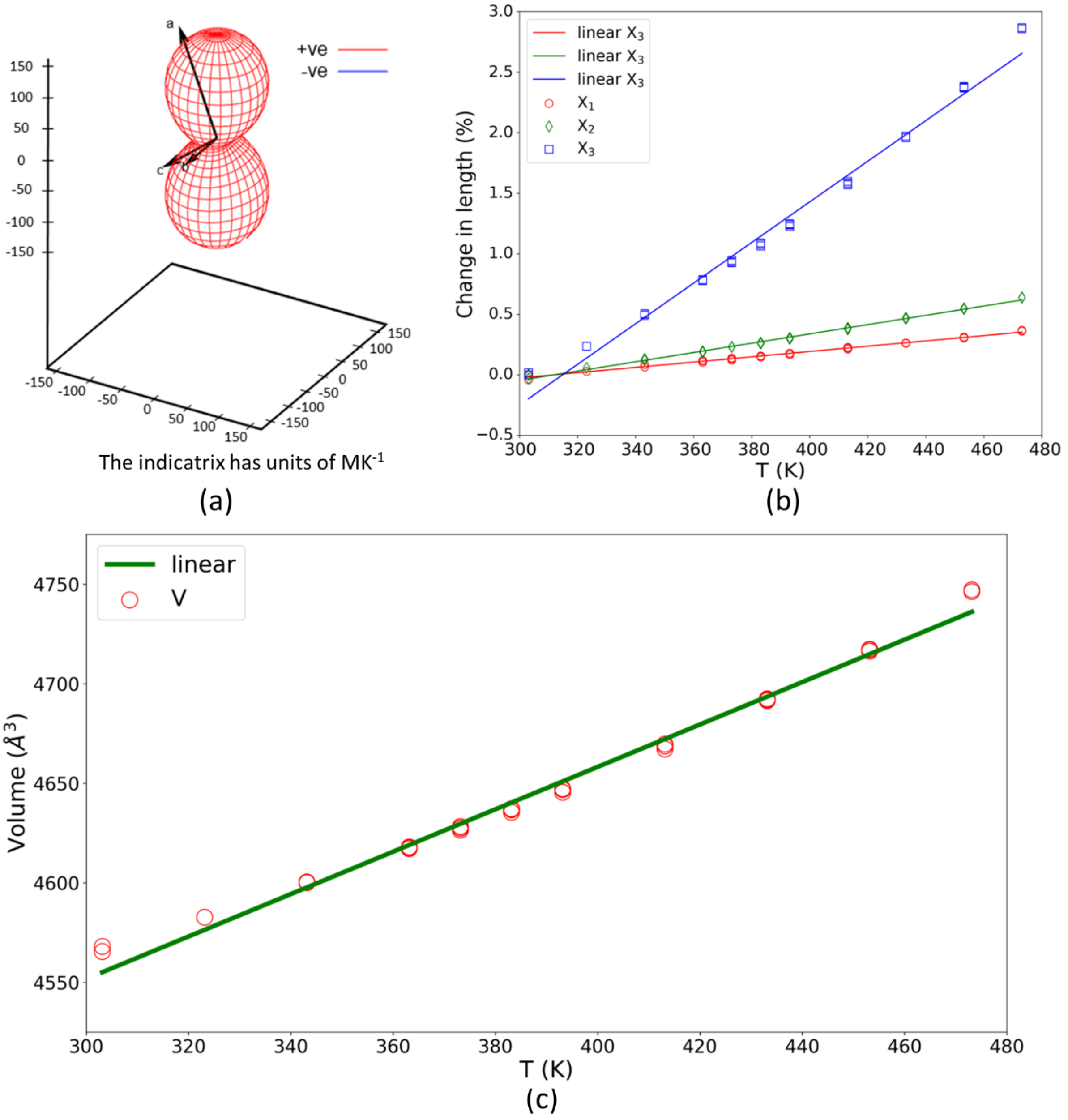

Figure S23 (a) A plot showing the variation of α with the direction, expansivity indicatrix, MK^{-1} in two different angles for **2**. (b) Overall thermal expansion of volume in **2**. (c) Anisotropic thermal expansion of volume in **2**.

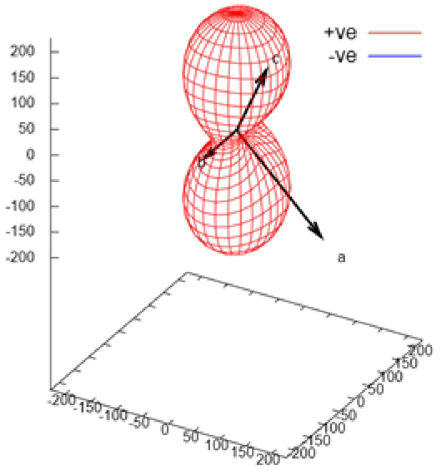

(a)

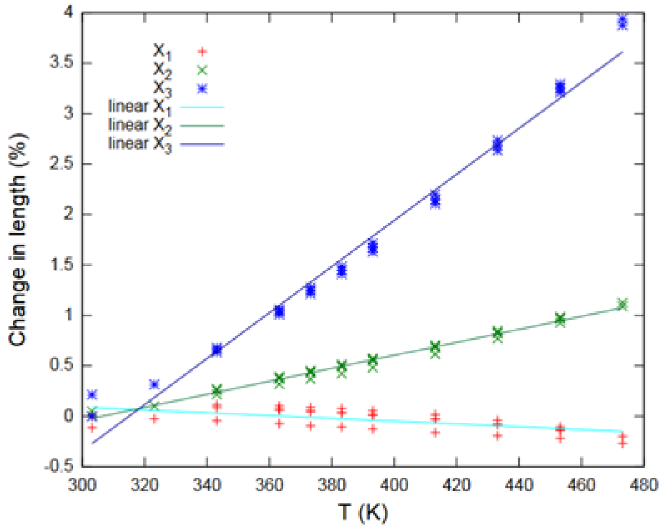

(b)

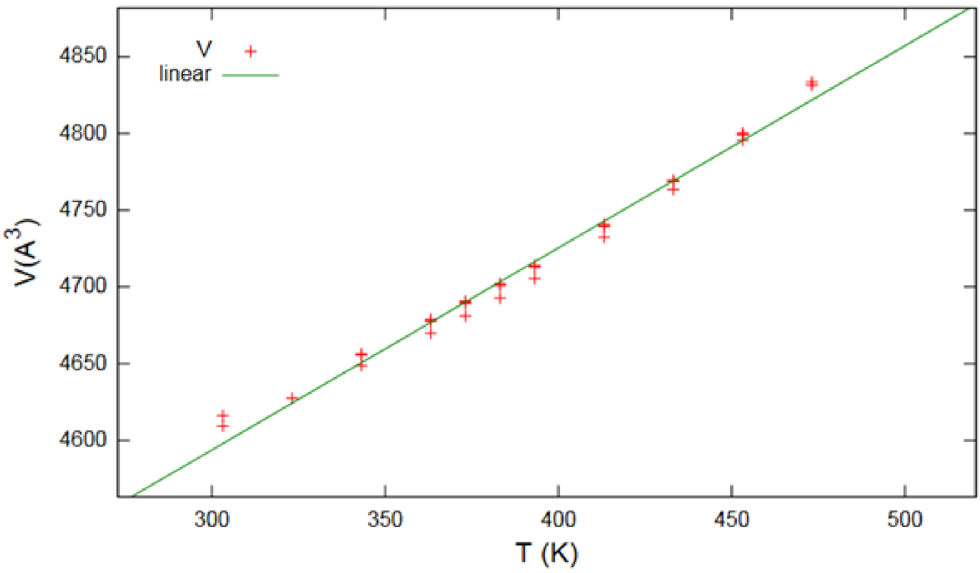

(c)

Figure S24 (a) A plot showing the variation of α with the direction, expansivity indicatrix, MK^{-1} in two different angles for **3**. (b) Overall thermal expansion of volume in **3**. (c) Anisotropic thermal expansion of volume in **3**.

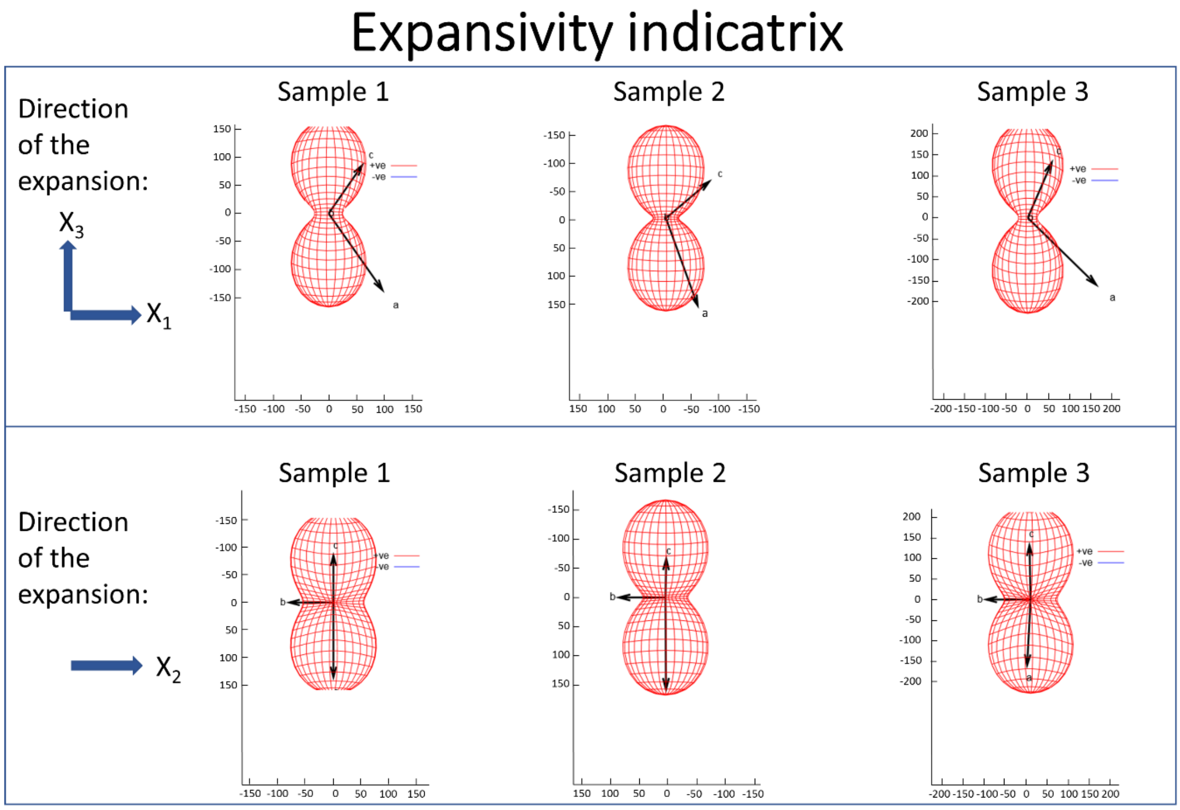

Figure S25 Overview of the expansivity indicatrix for Sample **1**, Sample **2** and Sample **3**.

10. Supplementary Tables

Table S1 Selected Crystallographic Data

	**1**	**2**	**3**	**5a**
Sp. Gr.	*C*2/*c*	*C*2/*c*	*C*2/*c*	$P\bar{1}$
a (Å)	24.633(11)	24.9699(8)	24.180(2)	10.3725(5)
b (Å)	12.093(4)	12.1074(4)	12.0004(9)	11.0398(5)
c (Å)	15.509(6)	15.4675(4)	15.598(1)	12.0294(5)
α (°)	90	90	90	71.354(1)
β (°)	108.48(1)	108.809(1)	108.802(2)	66.229(1)
γ (°)	90	90	90	65.356(1)
V (Å^3)	4381(3)	4426.4(2)	4461.7(5)	1126.61(9)
Z	4	4	4	1
D_{calc} (gcm^{-3})	1.477	1.516	1.504	1.489

Table S2 Changes in densities and volume in the photoreactions of **1-3**.

**Xtl**	**Density (exptl), g cm^{-3}**	**X-ray Density g cm^{-3}**	**Change in density (%)**	**Change in unit cell volume (%)**
**1**	1.48(2)	1.477	14.66	15.4
**4**	1.26(2)	NA		
**2**	1.50(2)	1.516	15.57	17.5
**5**	1.28(2)	1.489*		
**3**	1.50(2)	1.504	12.23	13.1
**6**	1.32(2)	NA		

* The sample was recrystallized and hence the discrepancy.

Densities of the compounds were measured by flotation method using hexane (density: 0.6548 g cm^{-3}) and tetrachloroethylene (TCE, density: 1.62 g cm^{-3}). V = (Z x MW) / (0.6022 x density)

Table S3 Some selected coefficients of uniaxial thermal expansion (TE) and volumetric thermal expansion (VTE) reported.[5]

**Compound**	**Temp. Range (K)**	**Uniaxial TE, $\alpha_x(10^{-6}\ K^{-1})$**	**VTE α_v (10^{-6} K^{-1})**	**Ref**
**Organic**				.
(S,S)-octa-3,5-diyn-2,7-diol	225-330	156<α_a>516<br>-32<α_b>-85<br>-48<α_c>-204		Ref [6]
18-crown-6-nitromethane	180-273	X_1 -129(15)<br>X_2 144(14)<br>X_3 282(16)	311	Ref [7]
**Co-crystal**				
4-aminobenzonitrile and 4-(dimethylamino) benzonitrile in 1:2	100-300	X_1 24.3<br>X_2 90.9<br>X_3 105	222	Ref [8]
1,2,3,4-cyclobutane-tetracarboxylic acid and<br>4,4′-bipyridylethylene	120-298	X_1 4(5)<br>X_2 25(4)<br>X_3 147(8)	183<br>(Monoclinic)	Ref [9]
4-phenylazopyridine<br>4,6-diCl and 4,6-dichlororesorcinol in 2:1	290-260	X_1 -116<br>X_2 29<br>X_3 316	229	Ref [10]
2(4PAzP)·(4,6-diBr res)	170-250	X_1 -2(4)<br>X_2 9(3)<br>X_3 172(6)	249	Ref [11]
**MOFs**				
[Cu_3(btc)$_2$] (btc =1,3,5-benzenetricarboxylate)	80-500		-4.1(1)	Ref [12]
FMOF1	295-90	230	300	Ref [13]
MCF-82 **1**<br><br>**1**-DMF<br><br>**1**-DMA	112-300	X_1 61(1)<br>X_2 482(12)<br>X_3 -218(3)<br>X_1 171(4)<br>X_2 60(3)<br>X_3-56(2)<br>X_1 85(1)<br>X_2 103(4)<br>X_3 -51(3)	319(13)<br><br>175(2)<br><br>138(2)	Ref [14]
**Metal Frameworks**				
$Ag_3[Co(CN)_6$	10-500	150	160	Ref [15]
**Metal Complexes**				
(phenylazophenyl)Pd(hexafluoroacetylacetonate)	220-350<br>100-270	260.4, 39.4, -79.9<br>124.0, 105.4, 114.9	247.8 (α form)<br>255.5 (β form)<br><br>213.13	Ref [16]

$[Cu(acac)_2]$	100-300	42, 44, 70		Ref [17]
**1**<br>**2**<br>**3**	300-480	13.9,<br>56.0, 166.4<br>21.9,<br>38.3, 167.8<br>-13.8,<br>64.5, 228.4	241.8<br>233.1<br>285.7	This work<br>This work<br>This work

11. Packing Fig.s

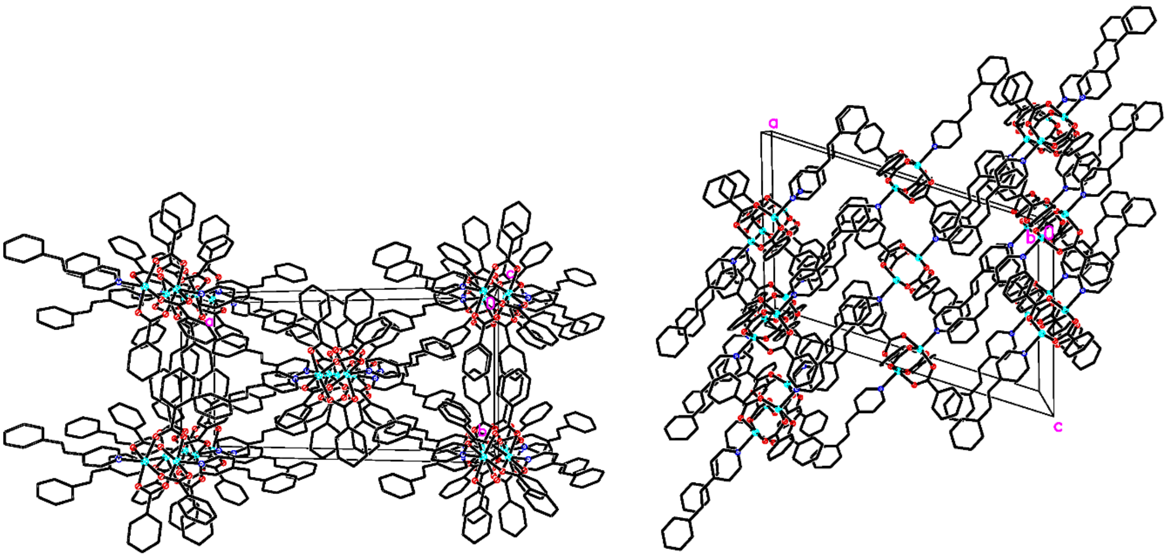

(a) **(b)**

Figure S26 Packing of **1** viewed down the *c*-axis (a) and (b) *b*-axis. The anisotropic expansion occurs approximately along *c*-axis.

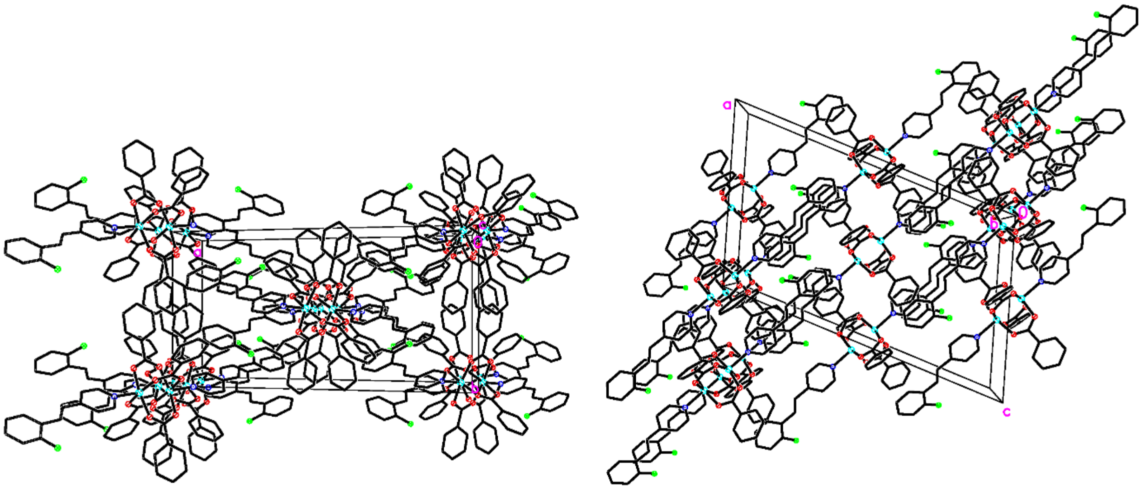

(a) **(b)**

Figure S27 Packing of **2** viewed down the *c*-axis (a) and (b) *b*-axis. The anisotropic expansion occurs approximately along *c*-axis.

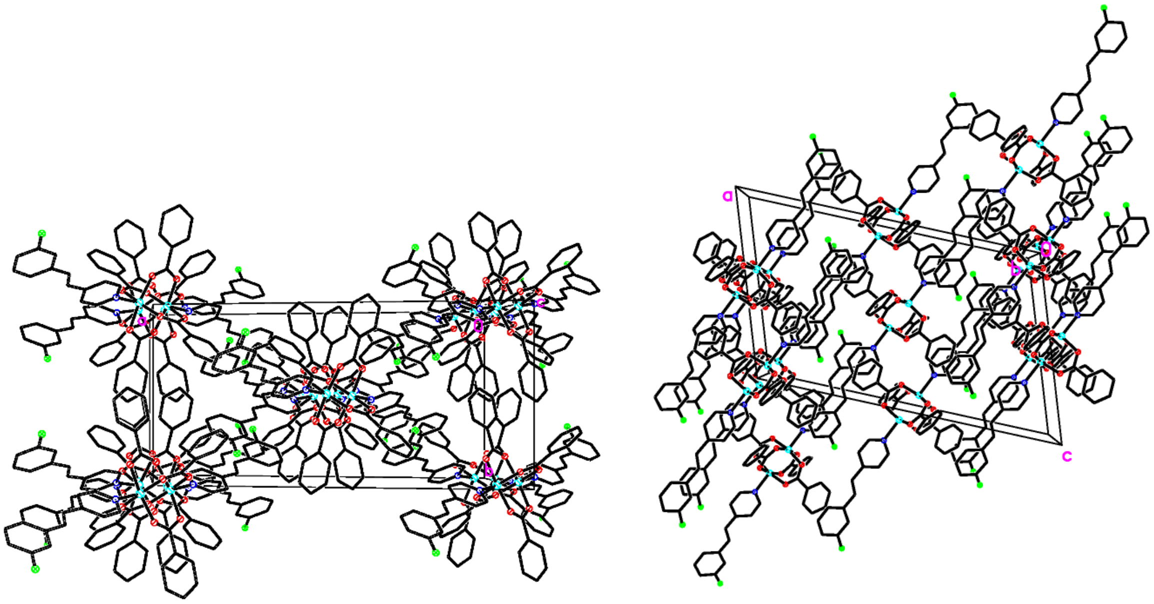

(a) **(b)**

Figure S28 Packing of **3** viewed down the *c*-axis (a) and (b) *b*-axis. The anisotropic expansion occurs approximately along *c*-axis.

12. Variable Temperature PXRD Experiments

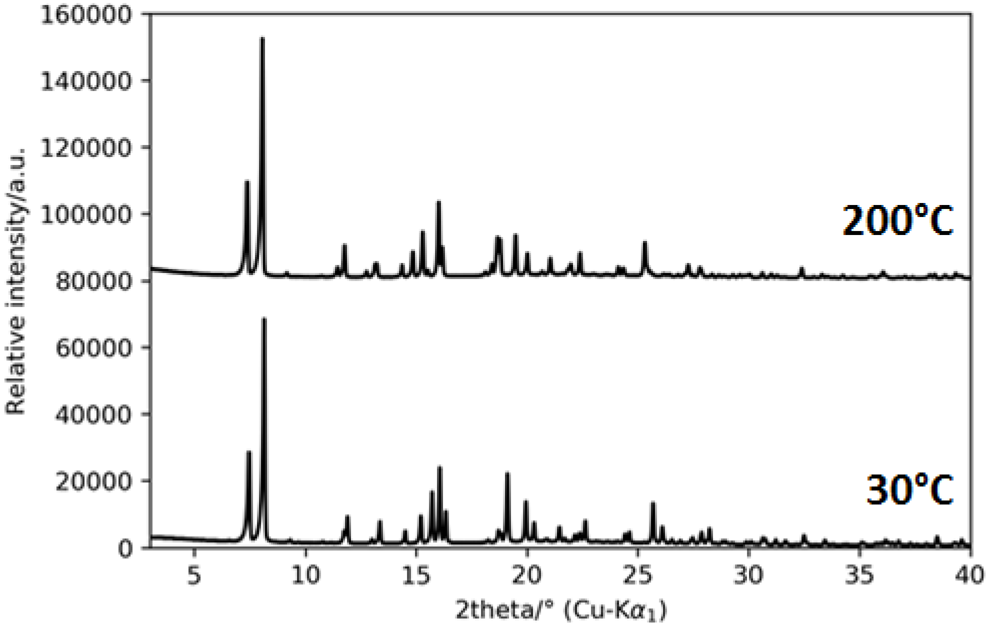

Fig. S29 PXRD patterns at two different temperatures for **1.**

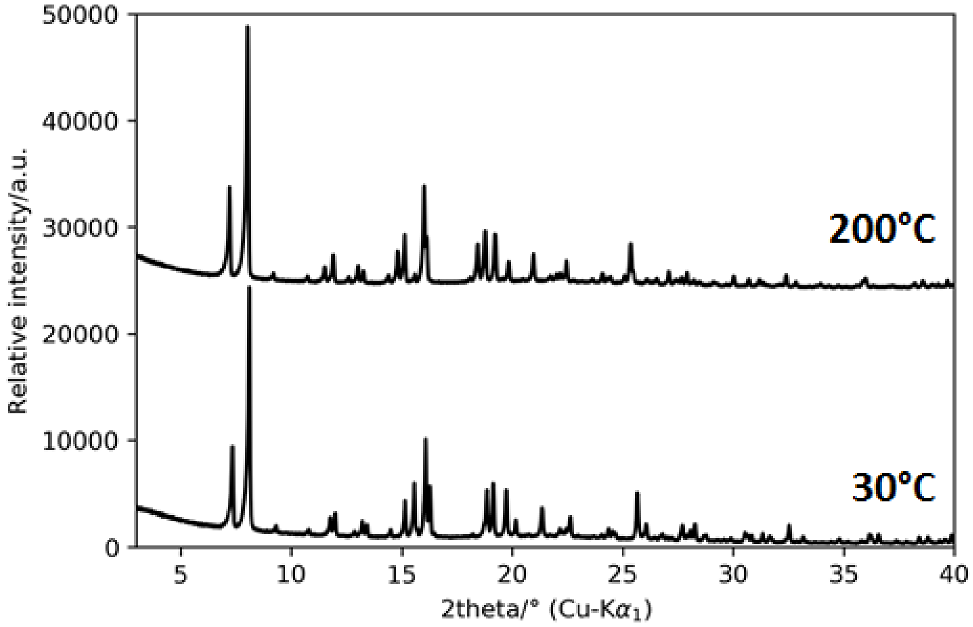

Figure S29 PXRD patterns after heating-cooling cycles for **2**.

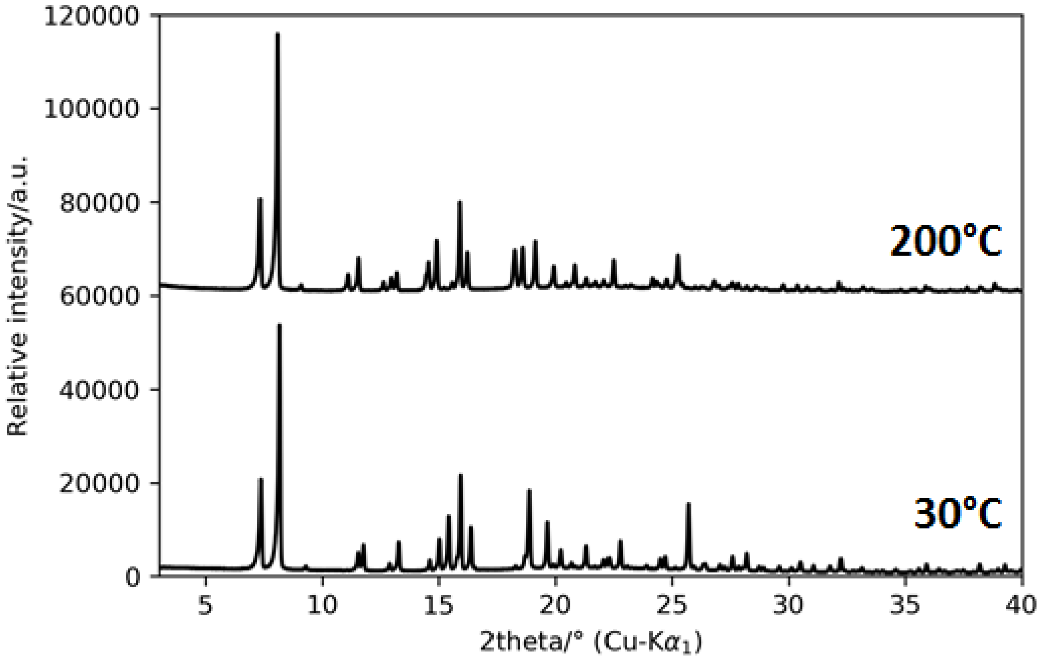

Figure S30 PXRD patterns at three different temperatures for **3**.

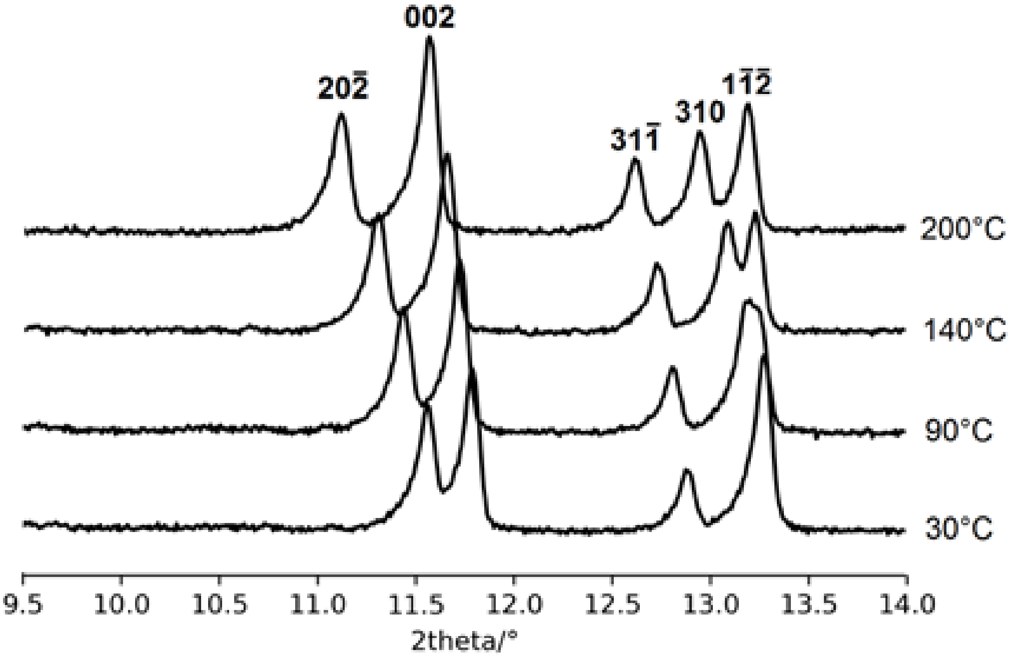

Figure S31 A typical PXRD pattern of **1** at three different temperatures showing the shifts in selected peaks related to the thermal expansion.

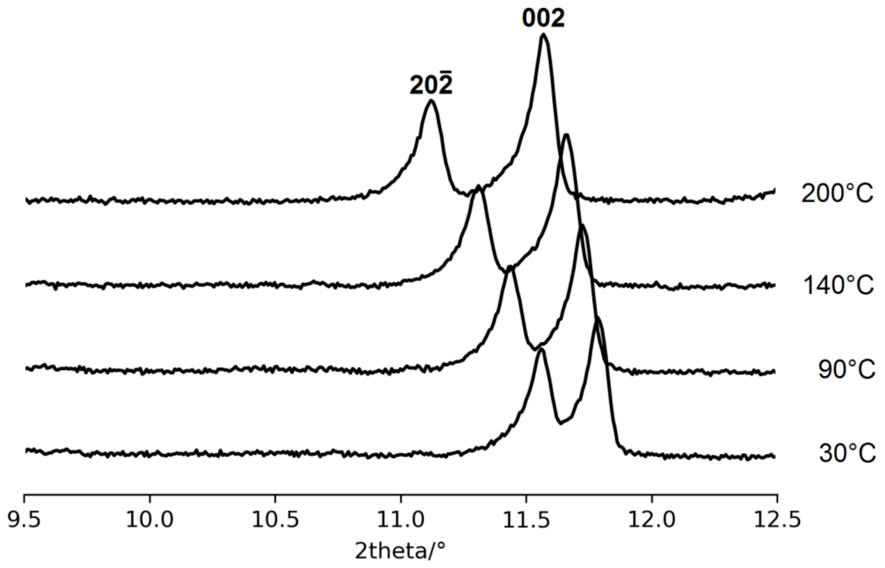

Figure S32 A typical PXRD pattern of **2** at three different temperatures showing the shifts in selected peaks related to the thermal expansion.

13. Determination of Volume and desities of 4, 5 and 6 by XRPD method

X-ray powder diffraction patterns were collected for compounds **4**, **5** and **6** (Fig.. S34). All compounds exhibit similar XRPD patterns which were indexed with triclinic unit cells (Table S4). In Fig.. S35, the plot of sample **4** was used as representative for all UV-irradiated compounds. The calculated density confirmed the trend of the experimental one with small values after UV irradiation (Table 5).

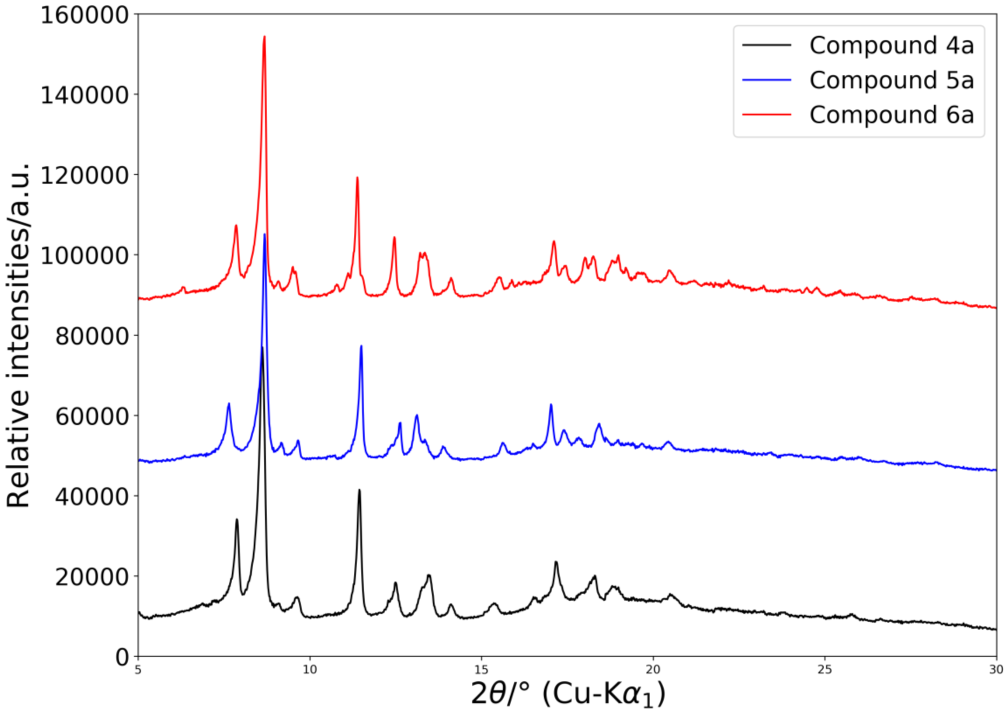

Figure S33 XRPD patterns of compounds **4** (black), **5** (blue) and **6** (red).

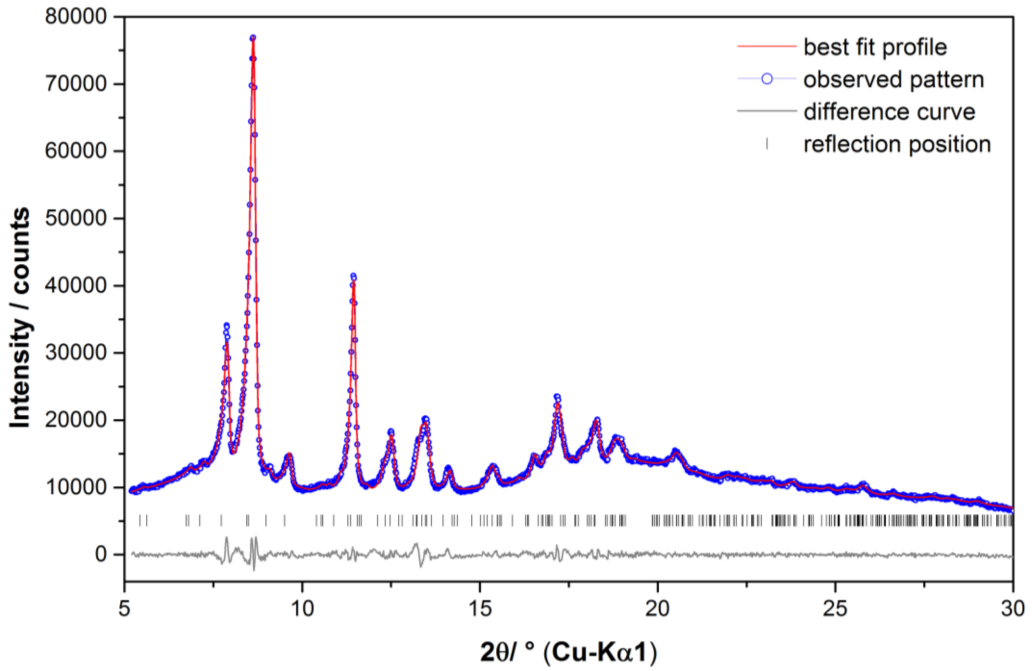

Figure S34 Pawley [18] fit using the trigonal unit cell to the data of Sample 4. The observed pattern (circles) measured in Debye-Scherrer geometry, the best fit profiles (line) and the difference curve between the observed and the calculated profiles (below) are shown.

Table S4 Crystallographic and Pawley Refinement data of the compounds **4**, **5** and **6** at ambient conditions.

**Compound**	**4**	**5**	**6**
**Crystal system**	Triclinic	Triclinic	Triclinic
**Space group**	$P\bar{1}(2)$	$P\bar{1}(2)$	$P\bar{1}(2)$
**Wavelength/ Å**	1.5406	1.5406	1.5406
***a* / Å**	14.317(3)	14.912(4)	14.344(3)
***b*/ Å**	17.037(5)	18.527(5)	16.949(4)
***c*/ Å**	16.420(6)	15.429(7)	16.343(6)
***α*/ °**	95.91(4)	93.28(2)	96.08(3)
***β*/ °**	98.39(2)	91.06(2)	98.10(2)
***γ*/ °**	112.68(4)	119.66(2)	111.50(2)
**V/ $Å^3$**	3600(20)	3692(20)	3606(20)
**T/ K**	298	298	298
**R_{wp}/% [a]**	2.57	3.00	4.23
**R_p/% [a]**	1.97	2.13	2.88
**Starting angle measured/ ° 2 ϑ**	0	0	0
**Final angle measured/ ° 2 ϑ**	110	110	110
**Starting angle used/ ° 2 ϑ**	5	5	5
**Final angle used/ ° 2 ϑ**	30	30	30
**Step width (° 2 ϑ)**	0.01	0.01	0.01
**Time(hrs)**	1	1	1

Table S5 Volume, calculated and experimental densities of the compounds **4, 5** and **6**. Since the solids showed high degree of disorder, high errors were used for the cell volume.

**Compound**	**4**	**5**	**6**
**V/ $Å^3$**	3600(20)	3692(20)	3606(20)
**Density (calc)**	1.30(1)	1.36(1)	1.39(1)
**Density (exp)**	1.26(2)	1.28(2)	1.32(2)
**Z**	2*3	2*3	2*3

14. List of Movies

Supplementary Movies 1-6 show PS Effect in **1-3**.

Supplementary Movies 7-12 show occasional and inconstant TS Effect in **1-3**.

16. References

1. S. N. Efange, R. H. Michelson, R. P. Remmel, R. J. Boudreau, A. K. Dutta, A. Freshler**,** Flexible JV-Methyl-4-phenyl-l,2,3,6-tetrahydropyridine Analogues: Synthesis and Monoamine Oxidase Catalyzed Bioactivation, J. Med. Chem., **1990**, *33*, 3133-3138.
2. G. M. Sheldrick, Program for Empirical Absorption Correction of Area Detector Data. *SADABS,* **1996**, *64*, 112.
3. G. Sheldrick, A short history of SHELX. *Acta Crystallogr. Sect. A* **2008**, *64*, 112–122.
4. S. M. R. Müller P., Herbst-Irmer R., Spek A. L., Schneider T. R., Crystal Structure Refinement: A Crystallographer's Guide to SHELXL, **2006**.
5. K. Yadava, Photoreactive and Photosalient Metal Complexes, Ph.D dissertation, Dept. of Chemistry, National University of Singapore, **2019**. ScholarBank@NUS Repository.
6. Das, D.; Jacobs, T.; Barbour, L. J. Exceptionally Large Positive and Negative Anisotropic Thermal Expansion of an Organic Crystalline Material. *Nat. Mater.* **2010**, *9*, 36–39.
7. Engel, E. R.; Smith, V. J.; Bezuidenhout, C. X.; Barbour, L. J. Uniaxial Negative Thermal Expansion Facilitated by Weak Host–guest Interactions. *Chem. Commun.* **2014**, *50*, 4238–4241.
8. Alimi, L. O.; Lama, P.; Smith, V. J.; Barbour, L. J. Large Volumetric Thermal Expansion of a Novel Organic Cocrystal over a Wide Temperature Range. *CrystEngComm* **2018**, *20* , 631–635.
9. Bhattacharya, S.; Saha, B. K. Interaction Dependence and Similarity in Thermal Expansion of a Dimorphic 1D Hydrogen-Bonded Organic Complex. *Cryst. Growth Des.* **2013**, *13* , 3299–3302.
10. Hutchins, K. M.; Groeneman, R. H.; Reinheimer, E. W.; Swenson, D. C.; MacGillivray, L. R. Achieving Dynamic Behaviour and Thermal Expansion in the Organic Solid State via Co-Crystallization. *Chem. Sci.* **2015**, *6*, 4717–4722.
11. Hutchins, K. M.; Kummer, K. A.; Groeneman, R. H.; Reinheimer, E. W.; Sinnwell, M. A.; Swenson, D. C.; MacGillivray, L. R. Thermal Expansion Properties of Three Isostructural Co-Crystals Composed of Isosteric Components: Interplay between Halogen and Hydrogen Bonds. *CrystEngComm* **2016**, *18*, 8354–8357.
12. Wu, Y.; Kobayashi, A.; Halder, G. J.; Peterson, V. K.; Chapman, K. W.; Lock, N.; Southon, P. D.; Kepert, C. J. Negative Thermal Expansion in the Metal–Organic Framework Material Cu_3(1,3,5-Benzenetricarboxylate)$_2$. *Angew. Chem. Int. Ed.* **2008**, *47*, 8929–893.
13. Yang, C.; Wang, X.; Omary, M. A. Crystallographic Observation of Dynamic Gas Adsorption Sites and Thermal Expansion in a Breathable Fluorous Metal–Organic Framework. *Angew. Chem. Int. Ed.* **2009**, *48*, 2500–2505.
14. Zhou, H.-L.; Zhang, Y.-B.; Zhang, J.-P.; Chen, X.-M. Supramolecular-Jack-like Guest in Ultramicroporous Crystal for Exceptional Thermal Expansion Behaviour. *Nat. Commun.* **2015**, *6*, 6917.
15. Goodwin, A. L.; Calleja, M.; Conterio, M. J.; Dove, M. T.; John, S. O.; Keen, D. A.; Peters, L.; Tucker, M. G.; Evans, J. S. O. Colossal Positive and Negative Thermal Expansion in the Framework Material $Ag_3[Co(CN)_6]$. *Science.* **2008**, *794*, 794–798.
16. Panda, M. K.; Runčevski, T.; Chandra Sahoo, S.; Belik, A. A.; Nath, N. K.; Dinnebier, R. E.; Naumov, P. Colossal Positive and Negative Thermal Expansion and Thermosalient Effect in a Pentamorphic Organometallic Martensite. *Nat. Commun.* **2014**, *5*, 1–8.
17. Brock, A.J.; Whittaker, J.J.; Powell, J.A.; Pfrunder, M.C.; Grosjean, A.; Parsons, S.; McMurtrie, J. C.; Clegg, J. K. *Angew. Chem. Int. Ed.* **2019**, *57***,** 11325–11328.
18. G. Pawley, J. Appl. Cryst. 14, 357–361 (1981).
